# Dietary intake and epigenetic aging in an African population: food groups, dietary patterns, and plant-based diets in the RODAM study

**DOI:** 10.1186/s12916-026-04884-y

**Published:** 2026-04-28

**Authors:** Naomi C.S. Auwerda, Muhau. M. Mungamba, Eva Van der Linden, Erik Beune, Karlijn A.C. Meeks, Peter Henneman, Charles Agyemang, Mary Nicolaou, Ina Danquah, Georges E. Janssens, Gajja S. Salomons, Felix P. Chilunga

**Affiliations:** 1 Department of Public and Occupational Health, Amsterdam Public Health Research Institute, Amsterdam University Medical Centers, University of Amsterdam, Amsterdam, The Netherlands; 2 Laboratory Genetic Metabolic Diseases, Amsterdam UMC, Amsterdam Gastroenterology, Endocrinology, and Metabolism, University of Amsterdam, Amsterdam Cardiovascular Sciences, Amsterdam, The Netherlands; 3 Division of Endocrinology, Diabetes and Nutrition, Department of Medicine, University of Maryland School of Medicine, Baltimore, MD USA; 4 Department of Human Genetics, Amsterdam UMC, University of Amsterdam, Amsterdam, The Netherlands; 5 Division of Endocrinology, Diabetes and Metabolism, Department of Medicine, The Johns Hopkins University School of Medicine, Baltimore, MD USA; 6 Faculty of Medicine and University Hospital, Heidelberg Institute of Global Health (HIGH), Heidelberg University, Heidelberg, Germany; 7 Department of Molecular Epidemiology, German Institute of Human Nutrition Potsdam-Rehbrücke (DIfE), Nuthetal, Germany; 8 Transdisciplinary Research Area (TRA) “Technology and Innovation for Sustainable Futures” and Center for Development Research (ZEF), University of Bonn, Bonn, Germany; 9 Amsterdam Gastroenterology, Endocrinology and Metabolism, Amsterdam, The Netherlands

**Keywords:** Epigenetic age acceleration, Dietary Patterns, Plant Based Food, Food groups, Sub-Saharan Africans.

## Abstract

**Background:**

Epigenetic age acceleration (EAA) is a biological marker of healthy longevity. While diet–EAA associations are documented in high-income countries, data from low- and middle-income populations—where diets can differ substantially—are unavailable. We examined associations between three complementary dietary components (specific food groups, dietary patterns, and plant-based food proportions) and EAA among Ghanaians across the nutrition transition (rural Ghana, urban Ghana, the Netherlands).

**Methods:**

We analyzed cross-sectional data from 705 adults in the RODAM study (2019–2021). Dietary intake was assessed via a Ghana-adapted Food Frequency Questionnaire (30 food groups; PCA-derived dietary patterns; Satija plant-based diet approach). EAA was estimated using DNA methylation clocks (Horvath, Hannum, PhenoAge, GrimAge) from Illumina EPIC array data. Linear regression models were used adjusted for demographic, lifestyle, health factors, and immune cell composition. Mediation analyses assessed potential biological pathways.

**Results:**

In rural Ghana, fish intake was associated with lower Horvath EAA (β= -0.028) and PhenoAge EAA (β= -0.026). In urban Ghana, fermented maize intake was inversely associated with Hannum EAA (β= -0.002) and PhenoAge EAA (β= -0.006). In Amsterdam, condiment intake was positively associated with Hannum EAA (β = 0.010) and PhenoAge EAA (β = 0.012). Dietary patterns and plant-based proportions were not associated with EAA. Dietary vitamins D, B9 and B12 partially mediated associations (11 to 49%).

**Conclusions:**

Among Ghanaians, intake of specific food groups (fish, fermented maize, condiments) were associated with EAA in specific contexts. Dietary patterns and plant-based food proportions did not show associations with EAA. Given the cross-sectional design, causal inference is not possible; longitudinal studies in these settings are warranted.

**Supplementary Information:**

The online version contains supplementary material available at 10.1186/s12916-026-04884-y.

## Background

Over the past three decades, global life expectancy has increased substantially [1]. However, gains in lifespan have not been matched by improvements in healthy longevity [1]. The burden of age-related chronic diseases has risen sharply; for example, the number of people living with type 2 diabetes increased from 150 to 430 million between 2000 and 2019 [2], while cardiovascular deaths rose from 14 to 18 million during the same period [3]. This widening gap between total and healthy life expectancy is increasingly concentrated in low- and middle-income countries (LMICs) [1], including in sub-Saharan Africa, where rapid demographic growth, urbanization, and epidemiological transition are driving sharp increases in cardiometabolic diseases alongside persistent infectious disease burdens [1–3]. Understanding determinants of healthy aging in these LMIC settings is therefore an urgent global health priority [1–3]. 

Unlike chronological aging, biological aging reflects the cumulative deterioration of molecular and cellular systems that drive vulnerability to disease, frailty, and ultimately, death [4]. This biological process can be quantified using DNA (Deoxyribonucleic acid) methylation-based epigenetic clocks [5], which estimate biological age by measuring methylation levels at specific Cytosine-phosphate-guanine (CpG) sites across the genome [5]. The difference between an individual’s epigenetic age and their chronological age, termed epigenetic age acceleration (EAA), has emerged as a robust biomarker of healthy longevity [5]. Elevated EAA, as measured by established epigenetic clocks such as Horvath [5], Hannum [6], PhenoAge [7], and GrimAge [8], consistently associates with a higher incidence of major age-related diseases, including cardiovascular disease and cancer (i.e. decreased healthy longevity) [5, 7, 8]. 

Among modifiable factors, diet has consistently emerged as a powerful determinant of EAA, particularly in studies conducted in high-income countries [7, 9–18]. Adherence to healthy dietary patterns, such as the Healthy Eating Index, Dietary Approaches to Stop Hypertension (DASH), Mediterranean diet, has been associated with lower PhenoAge EAA, GrimAge EAA, and DunedinPACE [13, 17–25]. Furthermore, controlled dietary interventions, including Mediterranean, caloric restriction and plant-based diets [16, 26], have demonstrated measurable reductions in EAA over time [16, 26]. Specific food groups are also implicated: higher intake of fish, leafy greens, and fruits is associated with slower EAA, while diets rich in red or processed meat, added sugars, and saturated fats show the opposite trend [7, 23]. 

Despite this compelling evidence, all existing studies on diet and EAA are from high-income populations [7, 9, 11–16, 19, 26]. This presents a critical gap in understanding how diet influences biological aging in populations exposed to vastly different food types, food patterns, cultural dietary norms, and stages of nutrition transition [27]. The nutrition transition refers to the shift from traditional, minimally processed diets to more energy-dense, processed diets high in fats and sugars that accompanies urbanization and economic development [27]. In LMICs, where rapid urbanization and adoption of Western lifestyles are underway, dietary composition ranges from predominantly traditional, minimally processed foods in rural areas to increasingly westernized diets that are often mixed with traditional foods in urban settings and among LMIC migrants in Europe [27–29]. These diverse diets differ in composition, quality, preparation methods, and nutrigenetic responses, and may lead to unique associations with EAA compared with those observed in high-income populations [22, 28]. For example, in many African settings, staple foods such as maize-based fufu or cassava-based equivalents are fermented, pounded, or boiled rather than refined or industrially processed [30]. These traditional methods powerfully influence starch structure, fiber content, and microbial composition, factors that could potentially enhance metabolic stability and significantly modulate the DNA methylation processes (the basis for calculating EAA) [5, 30]. Studying the unique diets of LMIC populations and their association with EAA is therefore essential: it will provide novel insight into how diet-specific factors in these contexts influence EAA and reveal how existing, culturally relevant foods and preparation methods can be strategically leveraged to promote healthy longevity.

To address this critical gap, we examined associations between three complementary diet components and EAA among Ghanaians across the nutritional transition spectrum (rural Ghana, urban Ghana, and The Netherlands). Specifically, we assessed [1] intake of key food groups (e.g., fish, fermented maize, green leafy vegetables, and sugar-sweetened beverages); [2] proportions of plant- and animal-based foods, as strict vegetarianism or veganism is uncommon in African populations; and [3] overall dietary patterns derived through principal component analysis.

## Methods

### Study population and design

Our study leverages data from the Research on Obesity and Diabetes among African Migrants (RODAM) cohort [31], a transcontinental, population-based study designed to investigate the interplay between genetic, environmental, and lifestyle factors in the development of obesity, type 2 diabetes, and other cardiometabolic disorders among Ghanaians living in different settings [31]. The study design and rationale have been described in detail previously [31]. In brief, the RODAM cohort recruited over 6,000 Ghanaian adults aged ≥ 18 years at baseline (2012–2015) across three geographic settings: rural Ghana (Ashanti region), urban Ghana (Kumasi and Obuasi), and European cities (Amsterdam, London, and Berlin). In Ghana, participants were selected through a multistage sampling strategy based on the 2010 national census, while in Amsterdam, Ghanaian adults were randomly sampled from the municipal registry using country-of-birth identifiers. In Berlin and London, recruitment was conducted through Ghanaian churches, community organizations, and social networks using a combination of respondent-driven and location-based sampling. Standardized protocols were applied across all sites to ensure harmonized data collection on sociodemographic characteristics, dietary intake, lifestyle factors, clinical measurements, and biological specimens [31]. 

A follow-up phase was conducted between 2019 and 2021, during which a subset of participants was reassessed using protocols comparable to those used at baseline [31]. This follow-up included participants from both rural and urban Ghana [31]. In Europe, however, follow-up data collection was limited to Amsterdam due to practical considerations such as funding constraints, logistical feasibility, and established infrastructure for participant tracking [31]. Harmonized procedures were maintained to ensure consistency in data collection across all follow-up sites [31]. 

For the current analysis, we performed a cross-sectional study using 2019–2021 data, as this point had larger Illumina EPIC 850 K DNA methylation data sample size than at baseline (follow-up: *N* = 718; baseline: *n* = 479) [31, 32]. The methylation subsample was selected based on hypertension status at follow-up to form a case-control sub-cohort [31]. Importantly, our previous research in this population showed no association between EAA and hypertension, supporting the use of this sample for EAA-related analyses [33]. Nonetheless, hypertension status was adjusted for in our analysis. Blood for DNA methylation measurement, dietary data, and covariates were collected at a single time point during a visit [31]. 

### DNA methylation and epigenetic aging

DNA methylation assessment in the RODAM study has been described in detail elsewhere [34]. In brief, whole blood samples were analyzed using the Illumina Infinium MethylationEPIC BeadChip, which quantifies methylation levels at over 850,000 CpG sites. Standard quality control procedures were followed (*minfi package in R*), including the exclusion of low-quality samples, individuals with sex mismatches, and probes that were cross-reactive, affected by common SNPs, or located on sex chromosomes [33]. Functional normalization was applied to minimize technical variation, and beta values (the proportion of methylation at each CpG site) were generated for downstream analysis [33]. 

Epigenetic age measures were derived using the Horvath Lab DNA Methylation Age Online Calculator (https://clockfoundation.org/), consistent with our previous work [33]. The calculator requires approximately 94,000 CpG probes and allows specification of Illumina EPIC array platform for accuracy. It generates a comprehensive output comprising more than 100 parameters, including epigenetic age estimates, EAA metrics, and estimated leukocyte cell-type proportions. The platform also flags cases in which required CpG probes are inadequate or missing and returns the corresponding outputs as not available (NA). In the RODAM dataset, approximately 93,000 required CpG probes overlapped with the reference panel and were successfully uploaded for analysis.

From the online epigenetic age calculator, we obtained estimates for four established DNA methylation–based aging clocks: HorvathAge, HannumAge, PhenoAge, and GrimAge. No warnings or missing outputs (NAs) were generated, indicating adequate CpG representation and robust age estimation. HorvathAge (353 CpGs) was designed as a multi-tissue predictor of chronological age [5], whereas HannumAge (71 CpGs) was trained specifically in whole blood [6]. PhenoAge (513 CpGs) integrates methylation sites associated with clinical biomarkers and mortality risk, thereby capturing phenotypic aging beyond chronological age [7]. GrimAge (1,030 CpGs) incorporates DNAm-based surrogates for plasma proteins and smoking pack-years and is optimized to predict morbidity and all-cause mortality [8]. 

Each clock captures a distinct biological dimension: HorvathAge reflects cellular aging and epigenetic maintenance; [5] HannumAge reflects immune-related aging; [6] PhenoAge reflects physiological and multi-system dysfunction; [7] and GrimAge reflects mortality risk [8]. Because these clocks provide complementary insights into overlapping aspects of health and longevity, all four were included in the analyses.

DunedinPACE was not included in this analysis. Although it can be applied to cross-sectional datasets, it measures the rate of biological aging rather than absolute cumulative biological age [35]. Given the inclusion of multiple epigenetic age measures across several dietary patterns and study sites, our interpretation focused on identifying consistent effect trends across clocks as a robustness check, as opposed to a single significant finding that may be a false positive. The inclusion of DunedinPACE, which operates on a fundamentally different biological scale and requires a distinct effect interpretation, would have complicated cross-clock comparability and the assessment of robustness.

EAA was calculated as the residual from regressing DNA methylation based age on chronological age. In the RODAM dataset, epigenetic clock estimates were strongly correlated with chronological age, confirming their validity in this study population (Additional file 1: Fig.S1). In previous publications, we also demonstrated that correlations among EAA measures were modest, ranging from *r* = 0.25 for Horvath EAA with GrimAge EAA to *r* = 0.62 for Hannum EAA with Horvath EAA, underscoring that these clocks capture overlapping but distinct biological dimensions of aging [33]. 

### Dietary assessment

Dietary assessment in the RODAM study has been described in detail elsewhere [29]. In brief, dietary intake was assessed using a semi-quantitative, Ghana-specific Food Propensity Questionnaire (Ghana-FPQ), designed to capture habitual food consumption in culturally relevant terms [29]. Reported foods were grouped into 30 predefined food categories based on culinary use and nutrient composition, including vegetables, fruits, legumes, whole and refined grains, fish, poultry, red meat, dairy, eggs, sweet spreads, sugar-sweetened snacks, and beverages (grams/day) [29]. 

Principal component analysis (PCA) with oblique rotation (ProMax; *psych package in R*) was used to identify data-driven dietary patterns based on the 30 food categories (Additional file 1: Figs.S2 and S3) [29]. Three major dietary patterns were retained based on eigenvalues, scree plot, and interpretability: [1] “traditional plant-based” pattern, rich in starchy staples, legumes, and vegetables [2] “mixed Western-Ghanaian” pattern characterized by high consumption of both traditional foods (staples, legumes) and Western items (meat, dairy, snacks); and [3] a modern/western “rice, meat, and fish” pattern, driven by high intakes of rice, poultry, red meat, and fish [29]. 

Additionally, a modified version of the Plant-Based Diet Index (PDI), adapted from Satija et al. (2016, Additional file 1.Table 1), was applied to quantify the relative intake of plant- and animal-based foods [36]. This modification was necessary because vegetarianism and veganism are rare among Ghanaians, making it infeasible to assess fully plant-based diets [29]. All foods were categorized into four overarching groups: healthful plant-based (e.g., fruits, vegetables, legumes, whole grains), unhealthful plant-based (e.g., refined grains, sweet spreads, sugar-sweetened snacks), animal-based (e.g., meat, dairy, eggs), and ambiguous (mixed-source or unclear items, Additional file 1: Table 2) [36]. For each participant, daily intake in grams/day was calculated for each group and expressed as a percentage of total food intake. To prevent collinearity in regression models, the ambiguous category was excluded from analysis (Additional file 1: Table 2) [36]. 

**Table 1:** Socio-demographic, lifestyle and anthropometric measures of Ghanaian RODAM participants (*n* = 705)

Variable	Rural Ghana (*n* = 259)	Urban Ghana (*n* = 208)	Amsterdam (*n* = 238)	*P*-value
**Chronological age (years)**	55 (45–63)	55(44–60)	55 (49–59)	0.033
Sex				0.008
*Male*	98 (37.8%)	62 (29.8%)	105 (44.1%)	
*Female*	161 (62.2%)	146 (70.2%)	133 (55.9%)	
Educational status				< 0.001
*Never been to school or elementary schooling*	155 (59.8%)	85 (40.9%)	76 (31.9%)	
*Lower secondary schooling*	82 (31.7%)	89 (42.8%)	101 (42.4%)	
*Higher secondary schooling*	16 (6.2%)	19 (9.1%)	48 (20.2%)	
*University*	6 (2.3%)	15 (7.2%)	13 (5.5%)	
Smoking status				0.174
*Yes*	11 (4.2%)	9 (4.3%)	8 (3.4%)	
*No. I have never smoked*	234 (90.3%)	189 (90.9%)	206 (86.5%)	
*No. but I used to smoke*	14 (5.4%)	10 (4.8%)	24 (10.1%)	
Physical activity ^a^				< 0.001
*Low level*	57 (22%)	63 (30.3%)	65 (27.3%)	
*Moderate level*	30 (11.6%)	75 (36.1%)	68 (28.6%)	
*High level*	172 (66.4%)	70 (33.7%)	105 (44.1%)	
BMI (kg/m^2^)	22.42 (19.7–25.89)	27.35 (23.80–32.42)	28.93 (25.86–31.79)	< 0.001
Diabetes mellitus ^b^				0.005
*No*	239 (92.3%)	183 (88.0%)	197 (82.8%)	
*Yes*	20 (7.7%)	25 (12.0%)	41 (17.2%)	
Hypertension ^c^				0.002
*No*	148 (57.1%)	102 (49%)	99 (41.6%)	
*Yes*	111 (42.9%)	106 (51%)	139 (58.4%)	

Data are presented as median (interquartile range, IQR), mean ± standard deviation (SD), or number and percentage, as appropriate. Physical activity was assessed using the WHO Global Physical Activity Questionnaire and categorized into low, moderate, or high levels. Diabetes mellitus was defined as self-reported physician diagnosis, use of glucose-lowering medication, or fasting blood glucose ≥ 7.0 mmol/L. Hypertension was defined as systolic blood pressure ≥ 140 mmHg, diastolic blood pressure ≥ 90 mmHg, or use of antihypertensive medication

**Table 2:** Dietary data among Ghanaian RODAM participants (*n* = 705) based on the Ghana-FPQ, per site

Dietary element	Rural Ghana (*N* = 259)	Urban Ghana (*n* = 208)	Amsterdam (*n* = 238)	*P*-value
	Median (IQR)	Median (IQR)	Median (IQR)	
**Specific food groups (grams/day)**				
Whole_grain_bread_cereals	2.7 (0–42.9)	24.5 (2.7–64.3)	53.2 (27.1–84.7)	< 0.001
White_bread_cereals	67.9 (47.2–150)	53.1 (32.9–107.1)	64.3 (25–103.7)	< 0.001
Sweet_spreads	0 (0–0)	0 (0–0)	0 (0–1.2)	< 0.001
Dairy_products	12.7 (5–37.1)	44.3 (17.5–100.4)	68.1 (20.4–156.8)	< 0.001
Fruit	213.7 (100.5–326.5)	172.4 (93.8–275)	313.7 (162.9–544.3)	< 0.001
Nuts_seeds	3.3 (1.3–9.9)	6.7 (2.7–17.1)	8.3 (1.7–22.7)	0.011
Roots_tubers_plantain	340.7 (215.9–517.9)	212.1 (150–298.4)	107.1 (64.6–160.5)	< 0.001
Potatoes	0 (0–0)	0 (0–10)	39.9 (13.3–64.3)	< 0.001
Fermente_maize_products	200 (120–280)	120 (69.3–200)	32.7 (18.7–69.3)	< 0.001
Vegetables	241.9 (168.7–317.4)	326.2 (261.6–401.4)	397.5 (273.1–529.3)	< 0.001
Legumes	57.1 (30–96.4)	57.1 (37.5–96.4)	36.7 (21.7–57.7)	< 0.001
Vegetable_soups_stews_sauces	302.9 (230.7–394.6)	313.6 (265.2–428.6)	200.5 (115.4–1015.3)	< 0.001
Rice_pasta	150 (66.8–166.1)	162.5 (91.5–227.7)	150 (71–192.9)	< 0.001
Egg	5 (2–12.9)	12.9 (5–30)	12.9 (5–12.9)	< 0.001
Red_meat	21.7 (10–50.5)	26.7 (10–52.9)	27.5 (10.6–60.9)	0.245
Poultry	5 (5–32.1)	12.5 (5–32.1)	37.5 (37.5–75)	< 0.001
Processed_meat	0 (0–0)	0 (0–11.7)	6.6 (0.7–17.8)	< 0.001
Fish	38.1 (23.3–56.2)	60.8 (42.9–88.7)	55.9 (31.7–77.3)	< 0.001
Meaty_mixed_dishes	64.3 (25–128.6)	64.3 (64.3–128.6)	50 (20–65.2)	< 0.001
Vegetarian_mixed_dishes	42.9 (21.4–42.9)	42.9 (21.4–42.9)	12.5 (5–32.1)	< 0.001
Cakes_sweets	14.2 (7.5–30.1)	10.5 (4.7–21.1)	15.5 (8.8–25.7)	< 0.001
Coffee_tea	0 (0–88.7)	85.7 (0–116.7)	591.1 (214.3–985.7)	< 0.001
Alcoholic_beverages	0 (0–8.3)	0 (0–0)	16.7 (0–73.9)	< 0.001
Sodas_juices	16.7 (6.7–32.5)	23.3 (14.6–54.2)	57.1 (13.3–150.6)	< 0.001
Palm_oil	1 (0.4–1.9)	1.1 (0.3–2.2)	0 (0–0.4)	< 0.001
Olive_oil	0 (0–0)	0 (0–0)	0.2 (0–1.5)	< 0.001
Other_oils	2.7 (1.3–4.7)	3.8 (1.9–7.3)	4.5 (1.1–19.6)	< 0.001
Margarine	0 (0–0)	0 (0–5.1)	2.5 (0–6)	< 0.001
Cooking_fats	0.1 (0.01–0.4)	0.2 (0.02–0.6)	0.4 (0.1–0.8)	0.099
Condiments	27.5 (6.7–56.2)	56.1 (30.4–77.5)	98 (63.2–146.8)	< 0.001
**Data driven dietary patterns (Scores)**				
DP1 (Plant-based & mixed traditional foods)	0.63 (0.03–1.45)	-0.61 (-0.94–-0.23)	-0.32 (-0.61–0.04)	< 0.001
DP2 (Animal-based and fried/staple-rich foods)	0.17 (-0.49–0.73)	-0.49 (-0.82–0.09)	-0.09 (-0.57–0.54)	< 0.001
DP3 (Modern/processed foods)	-0.80 (-1.27–-0.36)	0.40 (-0.17–1.04)	0.23 (-0.17–0.68)	< 0.001
**Proportion of plant/animal-based food intake (%)**				
Proportion healthful plant-based	48.21 (40.68–54.95)	41.99 ± 9.45	45.23 ± 12.86	< 0.001
Proportion unhealthful plant-based	21.87 (16.71–27.13)	20.34 (15.15–26.35)	18.99 (13.36–25.85)	0.003
Proportion animal-based	25.22 (20.11–31.36)	31.22 (25.2–39.27)	25.18 (19.17–37.09)	< 0.001
Proportion ambiguous	2.26 (1.12–3.82)	3.01 (1.97–4.47)	5.21 (3.5–6.98)	< 0.001

**Dietary intake** was assessed using semi-quantitative Food Frequency Questionnaires (FFQs), harmonized across study sites and adapted for Ghanaians. The table presents median and interquartile range (IQR) or mean ± standard deviation for intakes of **specific food groups** (in grams per day), data-driven dietary pattern (DP) scores, and proportions of plant- and animal-based food intake. **Dietary pattern** scores were derived using principal component analysis (PCA) with varimax rotation; higher scores indicate greater adherence to a given pattern. **DP1** reflects a plant-based and traditional mixed food pattern; **DP2** represents an animal-based and fried/staple-rich food pattern; and **DP3** captures a modern, processed food pattern. **Proportions of food intake** are expressed as percentages of total intake and categorized as healthful plant-based, unhealthful plant-based, animal-based, or ambiguous, based on a classification system adapted from Satija et al

### Other measurements

The full range of measurements in the RODAM study has been described elsewhere [31]. For this analysis, we included sociodemographic, lifestyle, clinical, and biological covariates relevant to aging and dietary research. Sociodemographic variables collected using structured questionnaires included chronological age in years, sex male or female, study site rural Ghana, urban Ghana, Amsterdam, and educational attainment no formal, primary, secondary, or tertiary education. Lifestyle factors included smoking status never, former, or current, physical activity low, moderate, or high assessed using the World Health Organization (WHO) Global Physical Activity Questionnaire, and body mass index (BMI ) in kg/m² [31]. Type 2 diabetes was defined by self-report, use of glucose-lowering medication, or fasting glucose 7.0 mmol/L or higher [31]. Hypertension was defined as systolic blood pressure 140 mmHg or higher, diastolic blood pressure 90 mmHg or higher, or use of antihypertensive medication [31]. 

Estimated blood immune cell proportions including B cells, CD4 + T cells, CD8 + T cells, granulocytes, monocytes, and NK cells were obtained as output from the online DNA methylation age calculator https://clockfoundation.org/, which applies the Houseman reference-based deconvolution method [37]. 

Dietary intake of vitamin B9 folate µg/day, vitamin B12 µg/day, and vitamin D µg/day was derived from the Ghana FPQ database given their roles in one carbon metabolism and antioxidant pathways relevant to DNA methylation and aging [29]. C-reactive protein (CRP) mg/L was measured using a high sensitivity radioligand binding assay as a marker of systemic inflammation [31]. Total energy intake kcal/day was calculated by summing the energy contributions of all reported foods using Ghana FPQ nutrient values and was used to derive Willett cut offs [31]. 

### Statistical analysis

All analyses were conducted using RStudio (version 4.4.1). Due to substantial contextual differences in diet and environment, analyses were stratified by study site: rural Ghana, urban Ghana, and The Netherlands. Missing data were less than 5% across socio-demographic, lifestyle, and health variables and were imputed using multiple imputation by chained equations (MICE; *mice package*). Descriptive statistics were used to summarize participant characteristics as means ± standard deviation, medians with interquartile ranges, or frequencies (%), as appropriate.

In the primary analyses, associations between EAA and 30 individual food groups were assessed using multivariable linear regression models. Collinearity among food groups was evaluated using the variance inflation factor (VIF) with a cut-off of 5 (*car package*); no collinearity was detected. All models were adjusted for chronological age, sex, education level, BMI, smoking status, physical activity, diabetes, and hypertension. Covariates were selected a priori based on established associations with both dietary exposures and epigenetic aging and were included to address potential confounding [20, 22, 25, 33]. Although EAA is derived from chronological age at the individual level, adjustment for chronological age remains necessary to address potential confounding across participants, as the derivation does not eliminate between-person age differences.

To enhance biological robustness across the four epigenetic clocks, a *“2-plus rule”* was applied. Associations were considered robust if the β-coefficient and its 95% confidence interval (CI) excluded zero in at least two clocks and showed a consistent direction across all four clocks within a study site. This approach is biologically motivated because the clocks capture distinct but overlapping aspects of cumulative biological aging; therefore, true associations are expected to replicate across multiple clocks rather than appear in isolation [33]. This method has been used in previous publications [33]. 

Additional primary models examined associations between EAA and [1] three PCA–derived dietary patterns (*psych package*), namely the plant-based and mixed traditional pattern, the animal-based and fried or staple-rich pattern *(“mixed Western-Ghanaian”)*, and the modern or processed pattern; and [2] dietary proportions of healthful plant-based, unhealthful plant-based, and animal-based food intake. These models used the same covariate adjustments and applied the 2-plus rule to identify robust findings, defined as statistically significant associations observed in at least two epigenetic clocks with consistent direction across all four clocks.

In secondary analyses, models were additionally adjusted for estimated leukocyte cell-type proportions. Because dietary exposures may influence EAA through inflammatory processes that alter immune cell abundance [38], adjustment for cell types was not performed in the primary analyses to avoid overcorrection of a potential biological pathway. Additional adjustment for estimated leukocyte proportions in secondary analyses was conducted to evaluate whether diet-EAA associations were independent of the immune cell pathway. All covariates in primary models were still retained.

Post hoc analyses were conducted to assess the robustness of findings from the primary and secondary analyses and to explore potential biological mechanisms. First, because the main results presented fully adjusted models due to the large number of analyses performed, stepwise regression models were additionally examined for statistically significant and robust findings. Models were sequentially adjusted for socio-demographic factors, lifestyle variables, clinical covariates, and immune cell composition to evaluate the stability of observed associations across incremental adjustment and to identify potential model-dependent effects. Second, analyses were restricted to participants with plausible energy intake, defined using Willett-recommended cut-offs of 500–3,500 kcal/day for women and 800–4,000 kcal/day for men [39]. Within this restricted sample, food group proportions were recalculated, dietary patterns were re-derived, and all regression models were repeated using the same covariate structure and predefined two-plus robustness criterion. Third, causal mediation analyses were conducted using the *mediation package* in R (1,000 bootstrap samples) to examine whether dietary intake of vitamin B9, vitamin B12, and vitamin D (derived from the Ghana-FFQ food composition database), as well as serum C-reactive protein, partially mediated the associations between dietary intake and EAA. Mediation was quantified as the proportion of the total effect explained by each biomarker.

## Results

### Participant inclusion

Of the 2,165 Ghanaian participants who took part in the RODAM follow-up study, a subset of 718 individuals with available 850 K DNA methylation data were selected for a nested case-control study on hypertension and DNA methylation. These 718 participants formed the basis of the present study. After excluding 13 individuals without dietary data, 705 participants remained for analysis. This final sample included 259 individuals from rural Ghana, 208 from urban Ghana, and 238 Ghanaian migrants residing in Amsterdam (Additional file 1: Fig.1).

### Baseline characteristics

Median chronological age was 55 years in rural Ghana, 52 years in urban Ghana, and 55 years in Amsterdam (Table 1). Women comprised the majority at all locations, representing 62% of participants in rural Ghana, 70% in urban Ghana, and 56% in Amsterdam. Educational attainment showed a clear gradient, with higher secondary or university education most prevalent in Amsterdam, intermediate in urban Ghana, and lowest in rural Ghana. Physical activity levels were highest in rural Ghana and substantially lower in urban Ghana and Amsterdam. The majority of participants across sites reported never smoking. Median BMI increased along a rural–urban–migrant gradient, lowest in rural Ghana and highest in Amsterdam. Diabetes and hypertension followed a similar pattern, with the lowest prevalence in rural Ghana, intermediate levels in urban Ghana, and the highest prevalence among migrants in Amsterdam (Table 1). Estimated immune cell proportions also varied by site (Additional file 1: Table 3). B-cell and CD8 + T-cell proportions were highest in rural Ghana and lower in urban Ghana and Amsterdam, whereas granulocyte proportions increased along the same rural–urban–migrant gradient. CD4 + T-cell, monocyte, and NK cell proportions were broadly comparable across sites.

**Table 3:** Epigenetic Aging Markers Among Participants (*N* = 705), by Study Site

Epigenetic aging marks	Rural Ghana (*n* = 259)	Urban Ghana (*n* = 208)	Amsterdam (*n* = 238)	*P*-value
	Mean (SD)/Median (IQR)	Mean (SD)/Median (IQR)	Mean (SD)/Median (IQR)	
**Epigenetic age (years)**				
*GrimAge*	59.33 ± 8.02	55.89 ± 8.61	56.40 ± 7.45	0.002
*PhenoAge*	48.13 ± 12.35	43.48 ± 10.13	43.03 ± 9.40	< 0.001
*Hannum*	47.52 ± 10.54	43.51 ± 8.29	43.16 ± 8.40	< 0.001
*Horvath*	55.13 ± 9.80	53.12 ± 8.13	54.61 ± 8.62	0.059
**Epigenetic age acceleration (years)**				
*GrimAge*	−0.63 (− 2.91–1.83)	−1.51 (− 3.60–0.31)	−2.61 (− 4.82–−0.48)	< 0.001
*PhenoAge*	2.24 (− 1.85–8.34)	−0.06 (− 4.21–4.98)	−2.38 (− 5.04–1.70)	< 0.001
*Hannum*	1.89 (− 1.99–6.00)	−0.08 (− 2.31–3.20)	−2.01 (− 4.87–1.51)	< 0.001
*Horvath*	0.66 (− 3.21–4.23)	0.41 (− 2.52–3.65)	0.62 (− 3.06–4.51)	0.984

Epigenetic age was estimated using four established DNA methylation-based clocks: GrimAge, PhenoAge, Hannum, and Horvath. Epigenetic age acceleration was calculated as the residuals from regressing epigenetic age on chronological age, indicating whether biological aging is faster (positive values) or slower (negative values) than expected. Data are reported as median (interquartile range, IQR) or mean ± standard deviation (SD), as appropriate

### Dietary intake characteristics

Intake of specific food groups differed across sites (Table 2). Intake of whole grains, dairy products, fruit, vegetables, nuts and seeds, poultry, processed meat, potatoes, coffee or tea, alcoholic beverages, sugar-sweetened beverages, olive oil, margarine, and condiments was lowest in rural Ghana, intermediate in urban Ghana, and highest in Amsterdam. In contrast, roots, tubers, plantain, and fermented maize products were most commonly consumed in rural Ghana. Vegetable-based soups and stews were more frequently consumed in Ghana. Fish intake was highest in urban Ghana, red meat intake was broadly comparable across sites, and poultry intake increased toward Amsterdam. Rice and pasta were consumed across all sites with modest variation, whereas sweet spreads and cooking fats were rarely consumed.

### Dietary patterns and plant/animal food proportions

Participants in rural Ghana most strongly adhered to the Plant-based & mixed traditional foods pattern (DP1) and the Animal-based and fried/staple-rich foods pattern (DP2; Table 2), whereas adherence to both patterns was lower in urban Ghana and Amsterdam. In contrast, participants in urban Ghana and Amsterdam more strongly adhered to the Modern/processed foods pattern (DP3) compared with those in rural Ghana (Table 2). Regarding plant and animal food proportions, rural Ghana had the highest proportion of healthful plant-based foods, accounting for approximately half of total intake, while this proportion was lower in urban Ghana and Amsterdam. Unhealthful plant-based foods were also relatively more common in rural Ghana, whereas animal-based food proportions were highest in urban Ghana (Table 2).

### Epigenetic age and acceleration

Epigenetic aging markers differed across study sites (Table 3). Mean epigenetic age was highest in rural Ghana for GrimAge, PhenoAge, and Hannum, with lower values in urban Ghana and Amsterdam, while Horvath age was almost comparable across locations. EAA patterns showed positive median values in rural Ghana for PhenoAge EAA (2.24 years), Hannum EAA (1.89 years), and Horvath EAA (0.66 years), and negative GrimAge EAA (–0.63). Urban Ghana had generally neutral median EAA values, while Amsterdam showed negative median EAA across GrimAge (–2.61), PhenoAge (–2.38), and Hannum (–2.01), with positive EAA only in Horvath (0.62; Table 3).

### Association between dietary intake and eaa (primary analyses, no immune cell adjustment)

Due to the large number of associations tested, only fully adjusted models were presented in primary analyses, controlling for age, sex, education, physical activity, smoking, BMI, hypertension, and diabetes (Tables 4 and 5). To obtain biologically robust findings from the four clocks, we applied a strict *“2 + rule*,*”* requiring that associations be statistically significant in at least two clocks with a consistent direction across all four.

**Table 4:** Association between specific food groups and epigenetic age acceleration (**Full adjusted primary analyses**)

Specific food groups	Horvath EAA	Hannum EAA	GrimAge EAA	PhenoAge EAA
	β (95%CI)	β (95%CI)	β (95%CI)	β (95%CI)
**Rural Ghana** (***n* = 259)**				
Alcoholic_beverages	-0.001 (-0.005–0.003)	0.002 (-0.002–0.006)	0.002 (0–0.004)	0.002 (-0.003–0.006)
Cakes_sweets	-0.022 (-0.055–0.011)	-0.021 (-0.054–0.012)	-0.009 (-0.027–0.009)	-0.038 (-0.08–0.003)
Coffee_tea	0.001 (-0.001–0.003)	0 (-0.002–0.003)	0 (-0.001–0.001)	0 (-0.003–0.003)
Condiments	-0.008 (-0.029–0.014)	-0.014 (-0.036–0.008)	-0.008 (-0.02–0.004)	-0.014 (-0.041–0.014)
Cooking_fats	-0.885 (-9.943–8.173)	-3.147 (-12.27–5.975)	-2.072 (-7.061–2.917)	-7.256 (-18.667–4.155)
Dairy_products	-0.01 (-0.028–0.007)	-0.015 (-0.033–0.002)	0.001 (-0.009–0.01)	-0.004 (-0.026–0.018)
Egg	-0.005 (-0.076–0.067)	-0.012 (-0.084–0.061)	0.01 (-0.03–0.049)	0.024 (-0.067–0.115)
Fermente_maize_products	0 (-0.005–0.005)	0.001 (-0.004–0.006)	0.001 (-0.002–0.003)	0 (-0.007–0.006)
Fish	-0.035 (-0.066–-0.004) *	-0.027 (-0.059–0.004)	-0.008 (-0.025–0.009)	-0.039 (-0.078–-0.003) *
Fruit	0.001 (-0.003–0.005)	0.001 (-0.003–0.005)	0.001 (-0.001–0.003)	0.001 (-0.004–0.006)
Legumes	-0.012 (-0.028–0.004)	0 (-0.017–0.016)	0.003 (-0.006–0.012)	-0.006 (-0.026–0.015)
Margarine	-0.149 (-0.424–0.126)	-0.141 (-0.418–0.136)	-0.108 (-0.259–0.044)	-0.012 (-0.36–0.336)
Meaty_mixed_dishes	-0.007 (-0.023–0.009)	-0.005 (-0.021–0.012)	-0.004 (-0.013–0.005)	-0.005 (-0.026–0.015)
Nuts_seeds	0.012 (-0.067–0.091)	-0.025 (-0.104–0.055)	0 (-0.043–0.044)	-0.001 (-0.101–0.098)
Olive_oil	-0.572 (-5.7–4.555)	-2.216 (-7.377–2.946)	0.846 (-1.98–3.672)	1.091 (-5.388–7.569)
Other_oils	0.012 (-0.058–0.081)	-0.001 (-0.071–0.069)	-0.023 (-0.061–0.015)	-0.021 (-0.109–0.067)
Palm_oil	0.284 (-0.077–0.644)	0.212 (-0.152–0.575)	-0.01 (-0.209–0.19)	0.357 (-0.098–0.812)
Potatoes	0.01 (-0.022–0.043)	0.002 (-0.03–0.035)	0.005 (-0.013–0.023)	0.013 (-0.028–0.054)
Poultry	0.013 (-0.032–0.058)	-0.023 (-0.068–0.022)	-0.006 (-0.031–0.018)	0.042 (-0.014–0.098)
Processed_meat	0.011 (-0.046–0.067)	0.007 (-0.05–0.063)	-0.022 (-0.053–0.009)	0.036 (-0.035–0.107)
Red_meat	-0.015 (-0.038–0.009)	-0.02 (-0.043–0.004)	-0.002 (-0.015–0.011)	-0.015 (-0.044–0.015)
Rice_pasta	-0.004 (-0.013–0.005)	-0.002 (-0.011–0.008)	0.002 (-0.003–0.007)	0 (-0.011–0.012)
Roots_tubers_plantain	0.001 (-0.002–0.003)	-0.001 (-0.003–0.002)	0 (-0.001–0.001)	0.001 (-0.002–0.004)
Sodas_juices	-0.001 (-0.005–0.003)	-0.001 (-0.006–0.003)	0.001 (-0.001–0.003)	0 (-0.005–0.006)
Sweet_spreads	0.419 (-1.715–2.553)	0.928 (-1.221–3.076)	0.461 (-0.715–1.637)	2.685 (0.009–5.361) *
Vegetable_soups_stews_sauces	-0.001 (-0.004–0.002)	0 (-0.003–0.003)	0.001 (-0.001–0.002)	0.001 (-0.003–0.004)
Vegetables	0.001 (-0.006–0.008)	0 (-0.007–0.007)	0.002 (-0.002–0.006)	-0.001 (-0.01–0.008)
Vegetarian_mixed_dishes	-0.017 (-0.06–0.026)	-0.007 (-0.05–0.036)	-0.007 (-0.03–0.017)	-0.02 (-0.075–0.034)
White_bread_cereals	-0.001 (-0.01–0.009)	0.004 (-0.006–0.013)	0.001 (-0.004–0.006)	-0.001 (-0.013–0.011)
Whole_grain_bread_cereals	0.002 (-0.015–0.019)	0.003 (-0.014–0.021)	-0.001 (-0.01–0.009)	0.004 (-0.018–0.026)
**Urban Ghana (** ***n*** ** = 208)**				
Alcoholic_beverages	0.013 (-0.005–0.031)	0.01 (-0.007–0.027)	0.007 (-0.004–0.019)	0.028 (0.005–0.052) *
Cakes_sweets	-0.028 (-0.075–0.019)	-0.021 (-0.066–0.024)	0.012 (-0.017–0.042)	-0.044 (-0.105–0.018)
Coffee_tea	0 (-0.002–0.002)	0.001 (-0.001–0.003)	0 (-0.002–0.001)	-0.002 (-0.005–0)
Condiments	-0.003 (-0.023–0.016)	-0.004 (-0.023–0.014)	0.005 (-0.007–0.017)	-0.014 (-0.04–0.011)
Cooking_fats	-14.734 (-34.118–4.65)	-13.9 (-32.351–4.55)	-3.697 (-15.823–8.43)	-19.634 (-45.13–5.863)
Dairy_products	-0.005 (-0.016–0.005)	-0.008 (-0.018–0.002)	0.004 (-0.002–0.011)	-0.019 (-0.032–-0.005) *
Egg	-0.042 (-0.097–0.013)	-0.053 (-0.105–-0.001) *	-0.009 (-0.044–0.025)	-0.07 (-0.142–0.002)
Fermente_maize_products	-0.002 (-0.006–0.001)	-0.004 (-0.007–-0.002) *	-0.002 (-0.004–0)	-0.007 (-0.012–-0.003) *
Fish	0.012 (-0.008–0.031)	0.008 (-0.01–0.027)	-0.001 (-0.013–0.011)	-0.016 (-0.042–0.009)
Fruit	0.002 (-0.003–0.008)	0.002 (-0.003–0.007)	-0.001 (-0.004–0.002)	-0.001 (-0.008–0.006)
Legumes	-0.004 (-0.022–0.013)	-0.009 (-0.025–0.007)	0.003 (-0.008–0.014)	-0.003 (-0.025–0.02)
Margarine	-0.024 (-0.243–0.194)	-0.071 (-0.279–0.137)	0.134 (-0.001–0.269)	-0.168 (-0.455–0.119)
Meaty_mixed_dishes	0.008 (-0.008–0.024)	0.003 (-0.012–0.018)	0.007 (-0.003–0.017)	0.004 (-0.017–0.025)
Nuts_seeds	0.027 (-0.036–0.09)	0.046 (-0.013–0.106)	0.049 (0.01–0.087)	0.031 (-0.052–0.113)
Olive_oil	0.235 (-0.714–1.183)	-0.137 (-1.041–0.766)	-0.251 (-0.841–0.339)	-0.453 (-1.7–0.794)
Other_oils	-0.06 (-0.123–0.003)	-0.031 (-0.092–0.029)	-0.003 (-0.043–0.036)	-0.003 (-0.087–0.08)
Palm_oil	-0.047 (-0.356–0.263)	0.035 (-0.26–0.33)	0.068 (-0.124–0.261)	-0.1 (-0.507–0.308)
Potatoes	-0.013 (-0.043–0.016)	0.007 (-0.021–0.035)	0.01 (-0.008–0.028)	-0.001 (-0.039–0.038)
Poultry	-0.003 (-0.043–0.037)	-0.003 (-0.041–0.036)	0.012 (-0.013–0.037)	-0.008 (-0.061–0.045)
Processed_meat	-0.016 (-0.063–0.032)	0.011 (-0.034–0.056)	0.03 (0.001–0.06)	0.023 (-0.039–0.085)
Red_meat	-0.01 (-0.031–0.011)	-0.022 (-0.042–-0.002)	0.012 (-0.001–0.025)	-0.017 (-0.045–0.011)
Rice_pasta	-0.003 (-0.011–0.005)	-0.006 (-0.014–0.002)	0.003 (-0.003–0.008)	-0.005 (-0.016–0.005)
Roots_tubers_plantain	0.002 (-0.004–0.007)	0.004 (-0.001–0.01)	0 (-0.003–0.004)	-0.003 (-0.01–0.004)
Sodas_juices	0.005 (0.001–0.01)	0.001 (-0.004–0.005)	-0.001 (-0.003–0.002)	0.001 (-0.004–0.007)
Sweet_spreads	0.255 (-0.157–0.667)	0.317 (-0.075–0.708)	-0.213 (-0.469–0.043)	-0.018 (-0.563–0.526)
Vegetable_soups_stews_sauces	-0.001 (-0.003–0.001)	-0.001 (-0.003–0.001)	-0.001 (-0.002–0)	0.001 (-0.002–0.003)
Vegetables	-0.001 (-0.008–0.005)	-0.002 (-0.008–0.005)	0 (-0.004–0.004)	-0.008 (-0.017–0.001)
Vegetarian_mixed_dishes	-0.007 (-0.05–0.035)	-0.03 (-0.07–0.01)	0.007 (-0.019–0.034)	-0.008 (-0.063–0.048)
White_bread_cereals	-0.001 (-0.01–0.007)	-0.001 (-0.01–0.007)	-0.001 (-0.006–0.005)	-0.002 (-0.013–0.009)
Whole_grain_bread_cereals	0.004 (-0.008–0.017)	0.004 (-0.008–0.016)	0.009 (0.001–0.016)	0.006 (-0.011–0.022) *
**Amsterdam (Ghanaians**,***n* = 238)**				
Alcoholic_beverages	-0.002 (-0.005–0.001)	-0.001 (-0.003–0.002)	0.001 (0–0.003)	0.001 (-0.002–0.004)
Cakes_sweets	-0.053 (-0.09–-0.015)	-0.016 (-0.05–0.018)	0.017 (-0.003–0.037)	-0.029 (-0.071–0.013)
Coffee_tea	0 (-0.001–0.001)	0 (-0.001–0.001)	0 (0–0.001)	0 (-0.001–0.001)
Condiments	0.001 (-0.013–0.012)	0.011 (0.001–0.023) *	0.003 (-0.004–0.01)	0.016 (0.002–0.03) *
Cooking_fats	-1.884 (-5.035–1.266)	-1.06 (-3.886–1.766)	-2.363 (-4.02–-0.707) *	-1.805 (-5.313–1.704)
Dairy_products	-0.004 (-0.011–0.003)	-0.001 (-0.008–0.005)	0.002 (-0.001–0.006)	0.003 (-0.005–0.012)
Egg	-0.023 (-0.076–0.03)	-0.027 (-0.075–0.02)	-0.01 (-0.039–0.018)	-0.033 (-0.092–0.026)
Fermente_maize_products	-0.005 (-0.015–0.006)	0.002 (-0.007–0.011)	0.004 (-0.002–0.009)	0.005 (-0.006–0.017)
Fish	0.008 (-0.013–0.029)	0.002 (-0.017–0.021)	0.008 (-0.004–0.019)	0.016 (-0.008–0.039)
Fruit	0 (-0.003–0.002)	0 (-0.002–0.002)	-0.001 (-0.002–0.001)	0.001 (-0.002–0.004)
Legumes	-0.001 (-0.022–0.02)	-0.022 (-0.04–-0.003)	0.001 (-0.01–0.013)	0.005 (-0.018–0.029)
Margarine	0.022 (-0.111–0.156)	-0.016 (-0.136–0.104)	0.016 (-0.055–0.087)	-0.033 (-0.181–0.116)
Meaty_mixed_dishes	0.008 (-0.011–0.027)	0.009 (-0.008–0.025)	-0.005 (-0.015–0.005)	0.004 (-0.017–0.024)
Nuts_seeds	0.017 (-0.022–0.057)	0.023 (-0.012–0.059)	0.003 (-0.018–0.024)	0.022 (-0.022–0.067)
Olive_oil	-0.088 (-0.444–0.269)	-0.195 (-0.513–0.123)	-0.141 (-0.33–0.049)	-0.229 (-0.624–0.166)
Other_oils	0.001 (-0.038–0.04)	0 (-0.035–0.034)	-0.006 (-0.027–0.015)	-0.005 (-0.049–0.038)
Palm_oil	-0.8 (-1.851–0.251)	-0.13 (-1.076–0.816)	0.482 (-0.078–1.042)	-0.605 (-1.778–0.567)
Potatoes	-0.005 (-0.021–0.01)	-0.008 (-0.022–0.005)	0.001 (-0.007–0.01)	0.007 (-0.01–0.025)
Poultry	-0.009 (-0.037–0.02)	0.004 (-0.021–0.03)	-0.006 (-0.021–0.009)	0.007 (-0.025–0.038)
Processed_meat	-0.038 (-0.078–0.002)	0.001 (-0.035–0.037)	0.013 (-0.008–0.035)	-0.023 (-0.068–0.021)
Red_meat	0.005 (-0.017–0.027)	0.013 (-0.007–0.033)	0.001 (-0.01–0.013)	-0.006 (-0.031–0.019)
Rice_pasta	-0.004 (-0.012–0.005)	0 (-0.008–0.007)	0 (-0.005–0.004)	0.001 (-0.009–0.01)
Roots_tubers_plantain	-0.001 (-0.007–0.005)	0 (-0.006–0.005)	0.001 (-0.003–0.004)	-0.003 (-0.01–0.003)
Sodas_juices	-0.001 (-0.004–0.001)	0 (-0.002–0.002)	0.001 (0–0.002)	0.001 (-0.002–0.003)
Sweet_spreads	-0.068 (-0.22–0.083)	-0.095 (-0.231–0.04)	0.03 (-0.051–0.111)	0.022 (-0.147–0.191)
Vegetable_soups_stews_sauces	0.001 (-0.001–0.002)	0 (-0.001–0.002)	0.001 (0–0.002)	0.001 (-0.001–0.003)
Vegetables	0 (-0.005–0.004)	0 (-0.004–0.004)	0.001 (-0.002–0.003)	0.002 (-0.003–0.007)
Vegetarian_mixed_dishes	0.019 (-0.018–0.057)	0.03 (-0.003–0.064)	0 (-0.02–0.02)	0.049 (0.007–0.091)*
White_bread_cereals	-0.003 (-0.012–0.006)	0 (-0.008–0.007)	0.001 (-0.004–0.006)	0.006 (-0.004–0.016)
Whole_grain_bread_cereals	-0.002 (-0.016–0.012)	0.01 (-0.002–0.022)	0.002 (-0.006–0.009)	0.009 (-0.006–0.024)

This table presents the associations between intake of specific food groups (grams/day) and four measures of epigenetic age acceleration (Horvath EAA, Hannum EAA, GrimAge EAA, and PhenoAge EAA), stratified by study site (rural Ghana, urban Ghana, and Amsterdam). **Beta coefficients (β) and 95% confidence intervals (CI)** are derived from linear regression models fully **adjusted** for age, sex, education level, smoking status, physical activity, BMI, alcohol intake, diabetes status, and hypertension status. Positive β values indicate greater epigenetic age acceleration, while negative values suggest slower biological aging relative to chronological age. Asterisks (*) and bolded denote statistically significant associations based on the 95% confidence interval (*p* ≤ 0.05). Overall significance is determined using the “2 + rule”: an association is considered robust if at least two clocks show statistically significant associations and all four clocks show a consistent direction of effect

**Table 5:** Fully Adjusted primary analyses: Associations Between Dietary Patterns, Plant-Based and Animal-Based Food Proportions, and Epigenetic Age Acceleration Among Ghanaian Adults, Stratified by Study Site

Specific food groups		Horvath EAA	Hannum EAA	GrimAge EAA	PhenoAge EAA
		Adjusted β (95%CI)	Adjusted β (95%CI)	Adjusted β (95%CI)	Adjusted β (95%CI)
**Data driven dietary patterns**					
**Rural Ghana** (***n* = 259)**					
DP1 (Plant-based & mixed traditional foods)		0.344 (-0.888–1.576)	0.379 (-0.863–1.62)	0.319 (-0.359–0.998)	0.364 (-1.193–1.921)
DP2 (Animal-based and fried/staple-rich foods)		-0.732 (-1.618–0.153)	-0.876 (-1.766–0.015)	-0.323 (-0.812–0.166)	-0.61 (-1.732–0.512)
DP3 (Modern/processed foods)		-0.218 (-1.065–0.63)	0.09 (-0.765–0.944)	0.111 (-0.356–0.579)	-0.011 (-1.082–1.061)
**Urban Ghana** (***n* = 208)**					
DP1 (Plant-based & mixed traditional foods)		0.063 (-1.136–1.262)	0.272 (-0.869–1.412)	0.092 (-0.655–0.838)	-1.006 (-2.577–0.565)
DP2 (Animal-based and fried/staple-rich foods)		-0.310 (-1.18–0.561)	-0.624 (-1.448–0.200)	0.588 (0.052–1.124) *	-0.726 (-1.867–0.415)
DP3 (Modern/processed foods)		-0.258 (-1.143–0.628)	-0.572 (-1.412–0.268)	0.022 (-0.53–0.574)	-1.206 (-2.36–-0.053) *
**Amsterdam (Ghanaians**,***n* = 238)**					
DP1 (Plant-based & mixed traditional foods)		-0.081 (-0.754–0.593)	0.006 (-0.597–0.61)	0.064 (-0.295–0.424)	0.604 (-0.141–1.35)
DP2 (Animal-based and fried/staple-rich foods)		-0.383 (-1.107–0.34)	0.147 (-0.502–0.796)	0.178 (-0.208–0.564)	-0.136 (-0.943–0.67)
DP3 (Modern/processed foods)		0.236 (-0.824–1.296)	0.328 (-0.62–1.277)	0.269 (-0.295–0.834)	1.089 (-0.082–2.26)
**Proportion of plant/animal-based food intake**					
**Rural Ghana** (***n* = 259)**					
Proportion healthful plant-based		0.228 (–0.163 − 0.619)	0.342 (–0.051–0.735)	0.237 (0.023–0.451) *	0.452 (–0.041–0.945)
Proportion unhealthful plant-based	0.196 (–0.203–0.595)		0.363 (–0.038–0.764)	0.249 (0.030–0.468) *	0.460 (–0.043–0.963)
Proportion animal-based	0.195 (–0.207–0.598)		0.347 (–0.057–0.752)	0.252 (0.031–0.472) *	0.468 (–0.040–0.975)
**Urban Ghana** (***n* = 208)**					
Proportion healthful plant-based	0.140 (–0.268–0.547)		0.091 (–0.291–0.473)	–0.119 (–0.371–0.133)	0.183 (–0.350–0.716)
Proportion unhealthful plant-based	0.083 (–0.330–0.495)		–0.042 (–0.429–0.344)	–0.176 (–0.431–0.079)	0.064 (–0.476–0.603)
Proportion animal-based	0.086 (–0.311–0.483)		0.005 (–0.367–0.377)	–0.149 (–0.395–0.096)	0.157 (–0.362–0.677)
**Amsterdam (Ghanaians**,***n* = 238)**					
Proportion healthful plant-based	0.069 (–0.225–0.363)		–0.205 (–0.468–0.058)	0.020 (–0.136–0.176)	–0.077 (–0.405–0.251)
Proportion unhealthful plant-based	0.027 (–0.286–0.339)		–0.229 (–0.508–0.050)	0.051 (–0.114–0.217)	–0.060 (–0.409–0.289)
Proportion animal-based	0.073 (–0.216–0.361)		–0.208 (–0.465–0.050)	0.052 (–0.101–0.205)	–0.061 (–0.383–0.261)

This table presents the associations between data-driven dietary patterns, proportions of plant-based and animal-based food intake, and four measures of epigenetic age acceleration (Horvath EAA, Hannum EAA, GrimAge EAA, and PhenoAge EAA), stratified by study site (rural Ghana, urban Ghana, and Amsterdam). The values shown are beta coefficients (β) and 95% confidence intervals (CI), derived from linear regression models. Associations are shown after full adjustment (in brackets) for age, sex, education level, smoking status, physical activity, BMI, alcohol intake, diabetes status, and hypertension status. Positive β values indicate greater epigenetic age acceleration, while negative values reflect slower biological aging relative to chronological age. Asterisks (*) and bolded indicate statistically significant associations based on the 95% confidence interval. Overall significance is determined using the “2 + rule,” whereby an association is considered robust if at least two epigenetic clocks show statistically significant associations and all four clocks show a consistent direction of effect

Using this approach, only a few food groups met the threshold: in rural Ghana, higher fish intake was associated with lower Horvath EAA (β = − 0.035; 95% CI: − 0.066, − 0.004) and PhenoAge EAA (β = − 0.039; 95% CI: − 0.078, − 0.003); in urban Ghana, higher fermented maize intake was associated with lower Hannum EAA (β = − 0.004; 95% CI: − 0.007, − 0.002) and PhenoAge EAA (β = − 0.007; 95% CI: − 0.012, − 0.003). In Amsterdam, condiment intake was positively associated with higher Hannum EAA (β = 0.011; 95% CI: 0.001, 0.023) and PhenoAge EAA (β = 0.016; 95% CI: 0.002, 0.030; Table 4). No dietary patterns or plant- or animal-based food proportions met the 2 + rule in any setting (Table 5).

### Association between dietary intake and eaa (secondary analyses, immune cell adjustment)

Because dietary intake may influence EAA through immune-related pathways, leukocyte proportions were not included in the primary models to allow estimation of the total effect. In secondary analyses, immune cell proportions were additionally adjusted for on top of the primary covariates (Appendices 7 and 8). The robust associations observed for fish, fermented maize products, and condiment intake remained statistically significant after immune adjustment (Additional file 1: Table 4). No dietary patterns or plant- or animal-based food proportions met the predefined robustness criterion in the immune-adjusted models (Additional file 1: Table 5).

### Post hoc analyses (step-wise models, willet cutoff’s, and mediation)

Post hoc analyses were conducted to better understand the main findings. First, we examined stepwise models rather than relying solely on the fully adjusted model to assess the stability of associations across sequential covariate adjustment. Associations for fish, fermented maize, and condiments were present in crude or minimally adjusted models and remained directionally consistent after further adjustment for lifestyle, comorbidity, and immune cell composition, with only modest attenuation (Additional file 1: Table 6).

Second, we applied Willett cut-offs for implausible energy intake (based on Willett-recommended thresholds; *n* = 630) to assess robustness to extreme dietary reporting. Application of these cut-offs did not alter the primary findings for fish and fermented maize. However, in the restricted sample, the condiment association was no longer statistically significant, and an additional association with the unhealthful plant-based dietary pattern emerged. Specifically, in rural Ghana, higher unhealthful plant-based food proportion was associated with higher Horvath EAA (β = 0.053; 95% CI: 0.012–0.093) and GrimAge EAA (β = 0.073; 95% CI: 0.029–0.096), with a consistent positive direction across all four clocks (Additional file 1: Table 7). This association was no longer statistically significant after additional adjustment for immune cell composition (Additional file 1: Table 8), suggesting a model-dependent effect.

Third, we examined whether intake of nutrients related to one-carbon metabolism (vitamins B9 and B12), vitamin D as an antioxidant, and CRP as a biomarker of systemic inflammation mediated the associations between dietary intake and EAA. In rural Ghana, vitamin D and vitamin B12 intake partially mediated the inverse association between fish intake and EAA, accounting for 12–46% of the total effect. In urban Ghana, all mediation proportions were less than 10% for the fermented maize–EAA association. In Amsterdam, vitamin B9 partially mediated the positive association between condiment intake and EAA, accounting for 11–18% of the total effect (Additional file 1: Table 9).

## Discussion

### Summary of findings

We investigated cross-sectional associations between dietary intake and EAA among Ghanaians living in rural Ghana, urban Ghana, and the Netherlands using complementary dietary metrics (specific food groups, dietary patterns, and plant-based food proportions). Higher fish intake in rural Ghana and higher fermented maize intake in urban Ghana were associated with lower EAA, whereas higher condiment intake in Amsterdam was associated with higher EAA. Dietary patterns and plant-based food proportions did not meet the predefined robustness criterion. In energy-restricted analyses applying Willett cut-offs, a higher proportion of unhealthful plant-based foods was associated with higher EAA in rural Ghana, though this association was attenuated after adjustment for immune cell composition. Mediation analyses suggested potential partial contributions of vitamin D and B12 to the fish associations and vitamin B9 to the condiment associations.

### Discussion of key findings

To our knowledge, this is the first study to examine the association between dietary intake and EAA in populations from LMICs. Using three complementary (multi-dimensional) dietary measures—individual food groups, data-driven dietary patterns, and plant/animal-based food proportions—we examined how dietary exposures across rural, urban, and migrant Ghanaian populations associate with four validated epigenetic clocks. Our findings contribute novel evidence from African populations, which are often underrepresented in aging and nutritional epigenetics research. Moreover, our study was conducted in a middle-aged cohort, whereas most prior studies have focused on older adults [11, 15, 17, 19, 20, 22–26], thereby extending the evidence to midlife, which could allow for earlier interventions before older age.

In both primary and secondary analyses (immune cell adjustment included vs. not included), we found that higher intake of fish in rural Ghana and fermented maize products in urban Ghana were consistently associated with lower EAA across at least two clocks with a consistent direction across all four clocks—our definition of robustness. The inverse association between fish and EAA is consistent with studies from high-income settings [7, 13]. One potential biological pathway could relate to Omega-3 fatty acids [40]. Omega-3 fatty acids have been proposed to influence DNA methylation patterns through one-carbon metabolism and pro-inflammatory pathways, which may also be relevant to the biological aging process [40]. However, we did not measure omega-3 fatty acid levels in our dataset and were therefore unable to directly test this mechanism. Future studies in African populations should evaluate whether omega-3 status may explain the fish-EAA associations and whether it varies by food source or preparation, which may help explain the rural, urban and Europe differences.

In contrast, the observed inverse association between fermented maize product intake and lower EAA is novel. While fermented foods such as kimchi, yogurt, and kefir have been studied for gut health and inflammation in Western contexts [41], their link to epigenetic aging has rarely been explored. Fermented maize is a staple in Ghana (e.g., kenkey, banku) and may contain live microbes, bioactive peptides, and short-chain fatty acids, which can alter the gut microbiota [42][43]. A balanced microbiome has been shown to regulate systemic inflammation and modulate host epigenetics, including DNA methylation [44]. The urban-specific association may reflect differences in fermentation and product composition, which may influence microbial diversity, metabolite yield, and nutrient availability [45]. As with omega-3s, we could not assess whether microbial diversity or microbial metabolites mediate the observed effects of fermented foods on EAA. This is an important future direction, as diet–microbiome–epigenome interactions are emerging as a key axis in aging biology. Studies incorporating microbiome measurements will be critical to validate the role of fermented foods in epigenetic regulation across diverse settings.

We found that condiment intake in Amsterdam was associated with higher EAA—also a novel finding. Condiments such as bouillon cubes, seasoning powders, and ready-made sauces may be rich in sodium, saturated fats, added sugars, and artificial additives [46]. Excess sodium may be one of the explanatory biological mechanisms as it has been linked to oxidative stress, vascular dysfunction, and systemic inflammation—key mechanisms implicated in accelerated epigenetic aging [47]. However, we could not assess the role of excess sodium in the condiments-EAA relationship. Further research is needed to confirm whether condiment association with higher EAA may act via the effects of excess sodium.

These food-specific findings support the notion that not all dietary associations with biological aging are captured by broad dietary patterns. Indeed, we found no consistent associations between EAA and either principal component–derived dietary patterns or plant/animal food proportions. This contrasts with findings from high-income populations, where adherence to dietary patterns like the Mediterranean diet, DASH, and the Healthy Eating Index have been associated with slower biological aging [7, 9, 12–15, 19, 26, 29]. The discrepancy in our study may be due to the limited alignment between Ghanaian dietary structures and Western dietary pattern indices. For example, our traditional plant-based pattern included beneficial foods like legumes and vegetables, but also energy-dense staples with high glycemic loads, such as yam and cassava. Furthermore, the absence of strict vegetarians or vegans in our population may have limited our ability to fully capture the effects of plant-only diets, which have been shown to have negative associations with EAA in high-income cohorts [29]. 

In light of these limitations, we examined plant- and animal-based food proportions, based on the adapted Plant-Based Diet Index from Sajita et al.[36] However, no consistent associations were found. One potential reason may be that African diets often involve mixed dishes, making classification less precise. Additionally, food processing methods (e.g., deep-frying plantains or adding palm oil to stews) may affect the health impact of otherwise plant-based foods, which may attenuate the associations with EAA seen in other settings [48]. 

Another important secondary finding emerged in our sensitivity analysis using Willett-recommended energy intake cut-offs (500–3,500 kcal/day for women; 800–4,000 kcal/day for men). In the primary models without adjustment for leukocyte distribution, higher intake of unhealthful plant-based foods—comprising refined grains, sweet spreads, and sugar-sweetened snacks—was associated with increased EAA in rural Ghana. This aligns with prior literature linking higher unhealthful plant-based diet indices with an increased risk of type 2 diabetes and cardiovascular disease [36]. However, this association was attenuated to non-significance upon adjustment for cell-type proportions. These results may suggest that in this context, the unhealthful plant-based foods-EAA associations may be mediated through shifts in immune cell composition.

Our mediation analyses partly helped us to understand our findings. Dietary intake of vitamin B9, B12 and vitamin D intake partially mediated associations between diet and EAA, accounting for 11–46% of the observed effects. This may be consistent with their known roles in one-carbon metabolism (vitamin B9, B12) and antioxidant defense (vitamin D), both of which may relate to DNA methylation fidelity and epigenetic stability [49, 50]. Previous experimental and observational studies have shown that deficiencies in these nutrients are associated with global hypomethylation and accelerated epigenetic aging [33]. While these vitamins did not fully explain the observed relationships, they point to potential nutritional pathways that could be targeted in future intervention studies.

While our findings suggest that higher intake of fish and fermented maize products, and lower intake of condiments may be associated with lower EAA, the cross-sectional nature of this study prevents causal inference. To determine whether these foods are causally related to EAA, intervention studies are needed to test whether increasing their consumption leads to measurable changes in EAA. If confirmed, this would justify promoting specific, culturally relevant foods—such as fresh fish and traditionally fermented staples, and less condiments—as part of targeted strategies to advance healthy aging in LMICs. This approach may be more effective and context-appropriate than promoting broad dietary patterns, which may not align with local food systems, culinary practices, or population health needs.

### Strengths and Limitations

This study has several strengths. It is the first to link dietary intake with epigenetic aging in an LMIC population. A key strength is the inclusion of multiple geographic and nutritional contexts—rural Ghana, urban Ghana, and Ghanaian migrants in the Netherlands—capturing diverse stages of the nutrition transition. Another strength is the focus on a middle-aged cohort, whereas most prior studies have examined older adults, which could in the future allow for earlier interventions before old age. The use of a multi-dimensional dietary assessment (individual food groups, dietary patterns, and plant/animal food proportions), stratified analyses, and robustness checks using the 2-plus rule further reinforce the internal validity of our findings. Notably, the detected associations were consistent across four established epigenetic clocks that capture different aspects of biological aging, suggesting the observed pattern is unlikely to be a clock specific artefact. The association between unhealthful plant-based food intake and higher EAA was only evident after applying Willett-recommended energy intake cut-offs, emphasizing the value of restricting analyses to plausible dietary reporters. Additionally, the mediation analyses add some biological plausibility to our findings, indicating partial statistical mediation by intake of vitamins B9, B12, and D — nutrients known to be involved in one-carbon metabolism and epigenetic regulation.

However, several limitations should be acknowledged. First, the cross-sectional design precludes causal inference regarding the relationship between diet and EAA. Nevertheless, in LMIC populations where diet–EAA associations have not previously been examined, this study provides initial evidence that associations may differ from those observed in high-income populations and may help stimulate further research in underrepresented settings. Second, dietary data were self-reported and therefore subject to recall and classification errors, particularly in the context of complex mixed dishes. Third, although we explored biologically plausible pathways, we lacked direct measures of omega-3 fatty acid status and gut microbiome composition, both established modulators of diet–epigenome interactions and potential missing components of the proposed causal framework. Finally, generalizability is limited to Ghanaian populations and may not extend to other African or LMIC settings with different dietary patterns and environmental exposures.

## Conclusions

Among Ghanaians, intake of specific food groups (fish, fermented maize, condiments) was associated with EAA in specific contexts. Dietary patterns and plant-based food proportions did not show associations with EAA. Given the cross-sectional design, causal inference is not possible; longitudinal studies in these settings are warranted.

## Supplementary Information

Below is the link to the electronic supplementary material.

Supplementary Material 1: Additional file 1: Fig. S1. Correlation between Epigenetic ages and Chronological age

Supplementary Material 2: STROBE Statement—checklist of items that should be included in reports of observational studies

## Data Availability

The dataset supporting the conclusions of this article has been deposited in the Zenodo repository: Chilunga FP, Agyemang C. Dataset for: Dietary Intake and Epigenetic Aging in an African Population: Food Groups, Dietary Patterns, and Plant-Based Diets in the RODAM Study. Zenodo. https://doi.org/10.5281/zenodo.18697021 The data are subject to controlled access due to the inclusion of sensitive individual-level information, including health, lifestyle, and epigenetic (DNA methylation) data, which are protected under the General Data Protection Regulation (GDPR) and participant consent agreements. Public unrestricted sharing is therefore not permitted in order to safeguard participant privacy and confidentiality. Access may be requested within Zenodo platform or by contacting the RODAM Data Access Committee at Rodamstudycoordinator@amsterdamumc.nl Requests are reviewed by the RODAM steering committee and require submission of a research proposal and a signed data use agreement. Decisions are typically communicated within four weeks. A copy of the data use agreement is available from the RODAM Data Access Committee upon request.Data may only be used for the approved research purpose and may not be redistributed or used for commercial purposes, and access does not confer authorship rights.

## Abbreviations

BMI: Body mass index
CI: Confidence interval
CpG: Cytosine–phosphate–guanine
CRP: C–reactive protein
DASH: Dietary Approaches to Stop Hypertension
DNA: Deoxyribonucleic acid
DP: Dietary pattern
EAA: Epigenetic age acceleration
FFQ / FPQ: Food Frequency Questionnaire / Food Propensity Questionnaire
Ghana: FPQ–Ghana–adapted Food Propensity Questionnaire
LMICs: Low–and middle–income countries
MICE: Multiple imputation by chained equations
NK: Natural killer (cells)
PCA: Principal component analysis
PDI: Plant–Based Diet Index
RODAM: Research on Obesity and Diabetes among African Migrants
SNP: Single nucleotide polymorphism
VIF: Variance inflation factor
WHO: World Health Organization
