# Supplementary material for: Dietary intake and epigenetic aging in an African population: food groups, dietary patterns, and plant-based diets in the RODAM study
Source: BMC Med. 2026 Apr 28;24:357. doi: 10.1186/s12916-026-04884-y (PMC13270629; doi:10.1186/s12916-026-04884-y)
Supplement: Supplementary file 1 — Supplementary Material 1: Additional file 1: Fig. S1. Correlation between Epigenetic ages and Chronological age [file 12916_2026_4884_MOESM1_ESM.pdf]

Additional file 1: Fig. S1. Correlation between Epigenetic ages and Chronological age

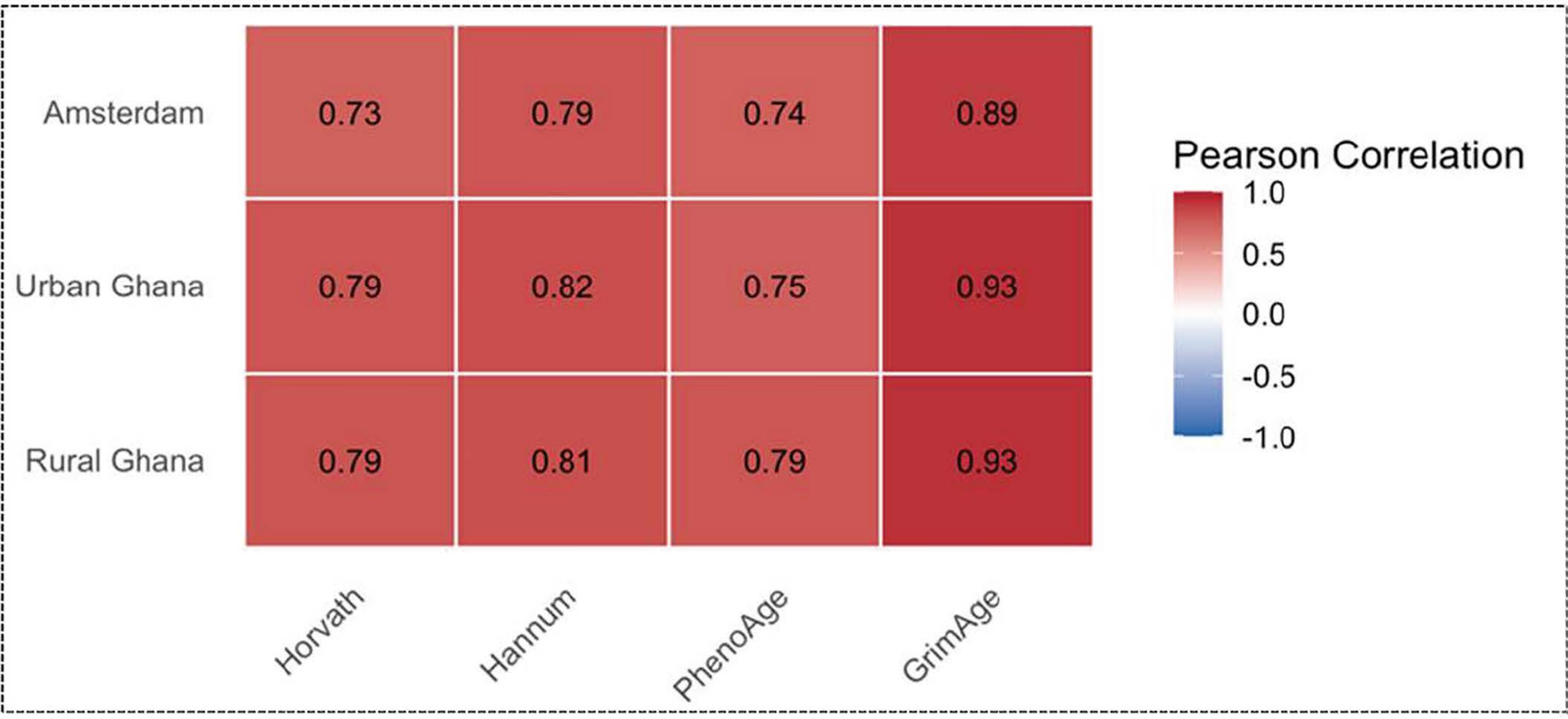

Additional file 1: Fig. S2. Factor Loadings for First 3 Dietary Patterns (PCs)

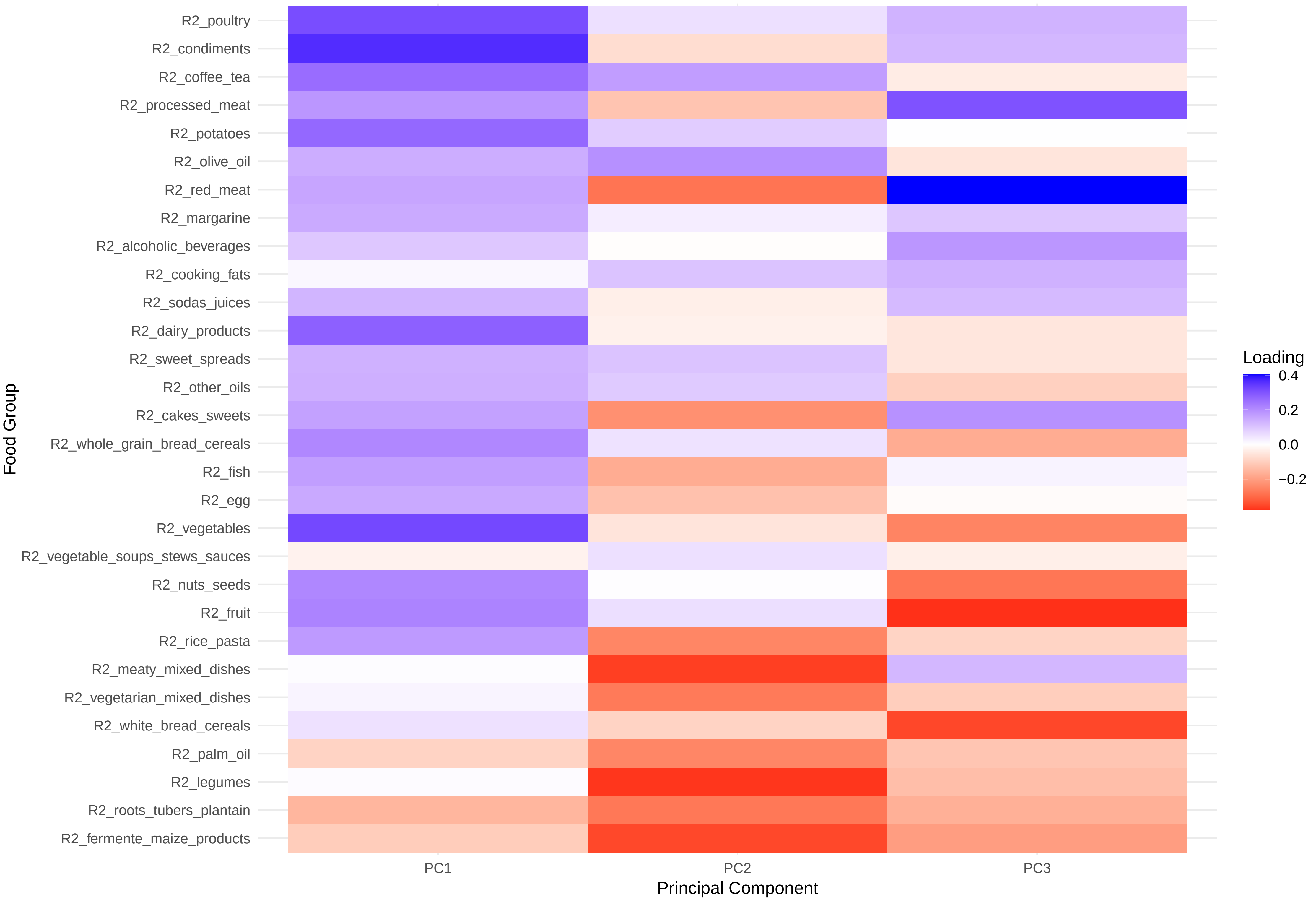

Additional file 1: Fig. S3 : Scree Plot: Variance Explained by Principal Components

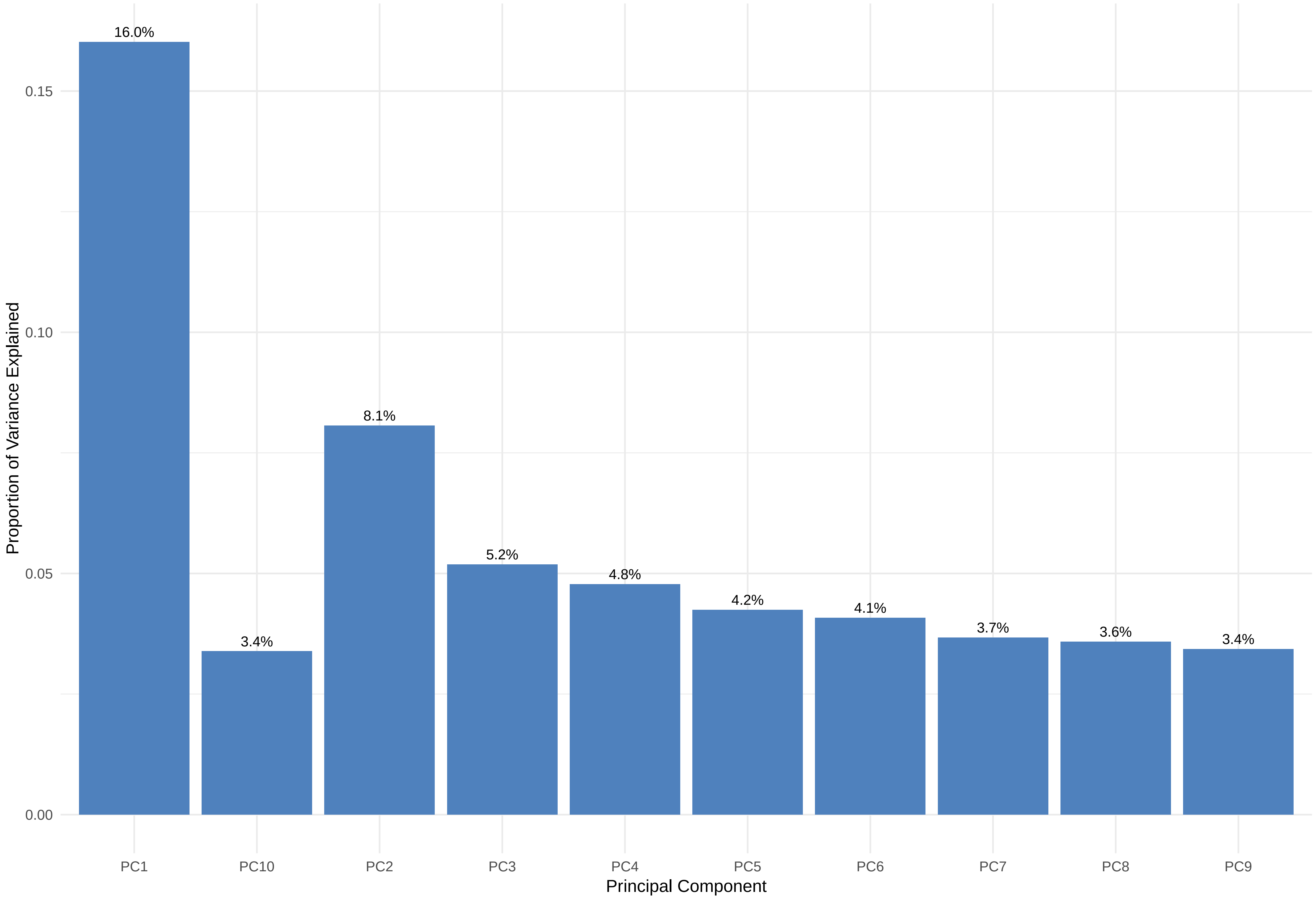

Additional file 1: Table 1. Categorization of healthy, less healthy, and animal food groups from Satija et al. (2016).

S1 Table. Examples of food items constituting the 18 food groups (from the 1984 NHS food frequency questionnaire)

Plant Food Groups	
Healthy	
**Whole grains**	Whole grain breakfast cereal, other cooked breakfast cereal, cooked oatmeal, dark bread, brown rice, other grains, bran, wheat germ, popcorn
**Fruits**	Raisins or grapes, prunes, bananas, cantaloupe, watermelon, fresh apples or pears, oranges, grapefruit, strawberries, blueberries, peaches or apricots or plums
**Vegetables**	Tomatoes, tomato juice, tomato sauce, broccoli, cabbage, cauliflower, Brussels sprouts, carrots, mixed vegetables, yellow or winter squash, eggplant or zucchini, yams or sweet potatoes, spinach cooked, spinach raw, kale or mustard or chard greens, iceberg or head lettuce, romaine or leaf lettuce, celery, mushrooms, beets, alfalfa sprouts, garlic, corn
**Nuts**	Nuts, peanut butter
**Legumes**	String beans, tofu or soybeans, beans or lentils, peas or lima beans
**Vegetable oils**	Oil-based salad dressing, vegetable oil used for cooking
**Tea & Coffee**	Tea, coffee, decaffeinated coffee
Less healthy	
**Fruit juices**	Apple cider (non-alcoholic) or juice, orange juice, grapefruit juice, other fruit juice
**Refined grains**	Refined grain breakfast cereal, white bread, English muffins or bagels or rolls, muffins or biscuits, white rice, pancakes or waffles, crackers, pasta
**Potatoes**	French fries, baked or mashed potatoes, potato or corn chips
**Sugar sweetened beverages**	Colas with caffeine & sugar, colas without caffeine but with sugar, other carbonated beverages with sugar, non-carbonated fruit drinks with sugar
**Sweets and Desserts**	Chocolates, candy bars, candy without chocolate, cookies (home-baked & ready-made), brownies, doughnuts, cake (home-baked & ready-made), sweet roll (home-baked & ready-made), pie (home-baked & ready-made), jams or jellies or preserves or syrup or honey
Animal Food Groups	
**Animal fat**	Butter added to food, butter or lard used for cooking
**Dairy**	Skim low fat milk, whole milk, cream, sour cream, sherbet, ice cream, yogurt, cottage or ricotta cheese, cream cheese, other cheese
**Egg**	Eggs
**Fish or Seafood**	Canned tuna, dark meat fish, other fish, shrimp or lobster or scallops
**Meat**	Chicken or turkey with skin, chicken or turkey without skin, bacon, hot dogs, processed meats, liver, hamburger, beef or pork or lamb mixed dish, beef or pork or lamb main dish
**Misc. animal-based foods**	Pizza, chowder or cream soup, mayonnaise or other creamy salad dressing

Additional file 1: Table 2. Categorization of food groups based on the Galbete et al. (2017) and Satija et al. (2016).

Food group from the Ghana-FPQ	Categorization for this study	Rationale
Alcoholic beverages	Covariate	Like in Satija et al. the models were adjusted for alcoholic beverages.
Cakes and sweets	Ambiguous	Contains both plant-based sugars and animal products like eggs, milk. Listed as “Less Healthy Plant” group in Satija et al.
Coffee and tea	Excluded	Coffee and tea are from plants, typically consumed without milk or sugar in traditional contexts (e.g. regular coffee, decaffeinated coffee, black and green tea, and fruit and herbal tea). Though coffee and tea are part of a person’s diet, the beverages can be categorized under fluid intake and do not significantly contribute to caloric intake.
Condiments	Ambiguous	Includes mayonnaise (contains eggs), salad cream, and other sauces—some may be animal-based (e.g., creamy dressings) while others are plant-based (e.g., tomato ketchup).
Cooking fats	Animal-based	Includes lard and animal fats, consistent with “Animal fat” group in Satija et al.
Dairy products	Animal-based	Made from animal milk (e.g., cow’s milk, yogurt, cheese, cream), directly derived from animals.
Egg	Animal-based	Eggs are directly from animals (chickens), used in boiled, fried, or mixed dishes.
Fermented maize products	Unhealthful plant-based	Includes kenkey, banku—fermented but high in starch and low in micronutrients due to processing.
Fish	Animal-based	Includes dried, smoked, fried, or fresh fish (e.g., tilapia, mackerel, herrings); key protein sources in Ghanaian diets.
Fruit	Healthful plant-based	Raw or stewed fruits like banana, mango, pawpaw; naturally sweet and unprocessed. Aligns with “Healthy Plant” group in Satija et al.
Legumes	Healthful plant-based	Includes beans, lentils, groundnut soup. Aligns with “Healthy Plant” group in Satija et al.
Margarine	Unhealthful plant-based	Though plant-based in origin, it's highly processed, high in trans fats and considered less healthy.
Meaty mixed dishes	Animal-based	Mixed dishes that explicitly contain meat, e.g. fufuo with groundnut soup.
Nuts and seeds	Healthful plant-based	Includes dried fruits, nuts, and seeds. Aligns with “Healthy Plant Food” group in Satija et al.
Olive oil	Healthful plant-based	Plant-derived oil, rarely used in traditional cooking, but included in European-adapted diets. Aligns with “Healthy Plant Food” group in Satija et al.
Other oils	Healthful plant-based	Includes vegetable oils like sunflower or groundnut oil. Aligns with “Healthy Plant Food” group in Satija et al.
Palm oil	Healthful plant-based	Plant-derived oil, listed in “Palm oil” group and used in traditional Ghanaian cooking. Aligns with “Healthy Plant Food” group in Satija et al.
Potatoes	Unhealthful plant-based	Often deep-fried (e.g., chips) or eaten with sauces; more processed compared to yams or plantain. Listed as “Less Healthy Plant” group in Satija et al.
Poultry	Animal-based	Poultry is frequently consumed in stews, grilled, or fried forms.
Processed meat	Animal-based	Includes sausages, corned beef, and luncheon meat; used in stews or eaten with bread/rice. Heavily processed but still animal based.
Red meat	Animal-based	Includes beef, goat, pork, and bush meat—common in Ghanaian soups and grilled dishes.
Rice and pasta	Unhealthful plant-based	Usually white rice and instant noodles, often served with oil-heavy stews—refined and low in fiber. Listed as “Less Healthy Plant” group in Satija et al.
Roots, tubers and plantain	Healthful plant-based	Includes traditional staples like cassava, yam, plantain. Aligns with “Healthy Plant” group in Satija et al.
Sodas and juices	Unhealthful plant-based	Sugar-sweetened beverages, mostly plant-based but nutritionally poor. Listed as “Less Healthy Plant” group in Satija et al.

Sweet spreads	Unhealthful plant-based	Jams, honey, chocolate spreads—plant-derived sugars but low nutritional quality. Listed as “Less Healthy Plant” group in Satija et al.
Vegetable soups, stews and sauces	Animal-based	Though palmnut soup, nkontomire stew and okro stew are vegetable-based, traditional recipes often include meat/fish; Galbete et al. categorizes these under “Vegetable soups, stews, sauces,” but on the Ghanaian population level, these dishes are prepared with animal ingredients.
Vegetables	Healthful plant-based	Includes green leafy vegetables, tomatoes, etc. Aligns with “Healthy Plant” group in Satija et al.
Vegetarian mixed dishes	Healthful plant-based	Includes meatless dishes like red red and ampesie.
White bread and cereals	Unhealthful plant-based	Made from refined wheat flour (e.g., white bread, porridge), low in fiber, high glycemic index. Listed as “Less Healthy Plant” group in Satija et al.
Whole grain bread and cereals	Healthful plant-based	Includes brown rice, millet, whole grain bread—minimally processed and high in fiber. Aligns with “Healthy Plant” group in Satija et al.

Additional file 1: Fig. S4. Flow chart of participation

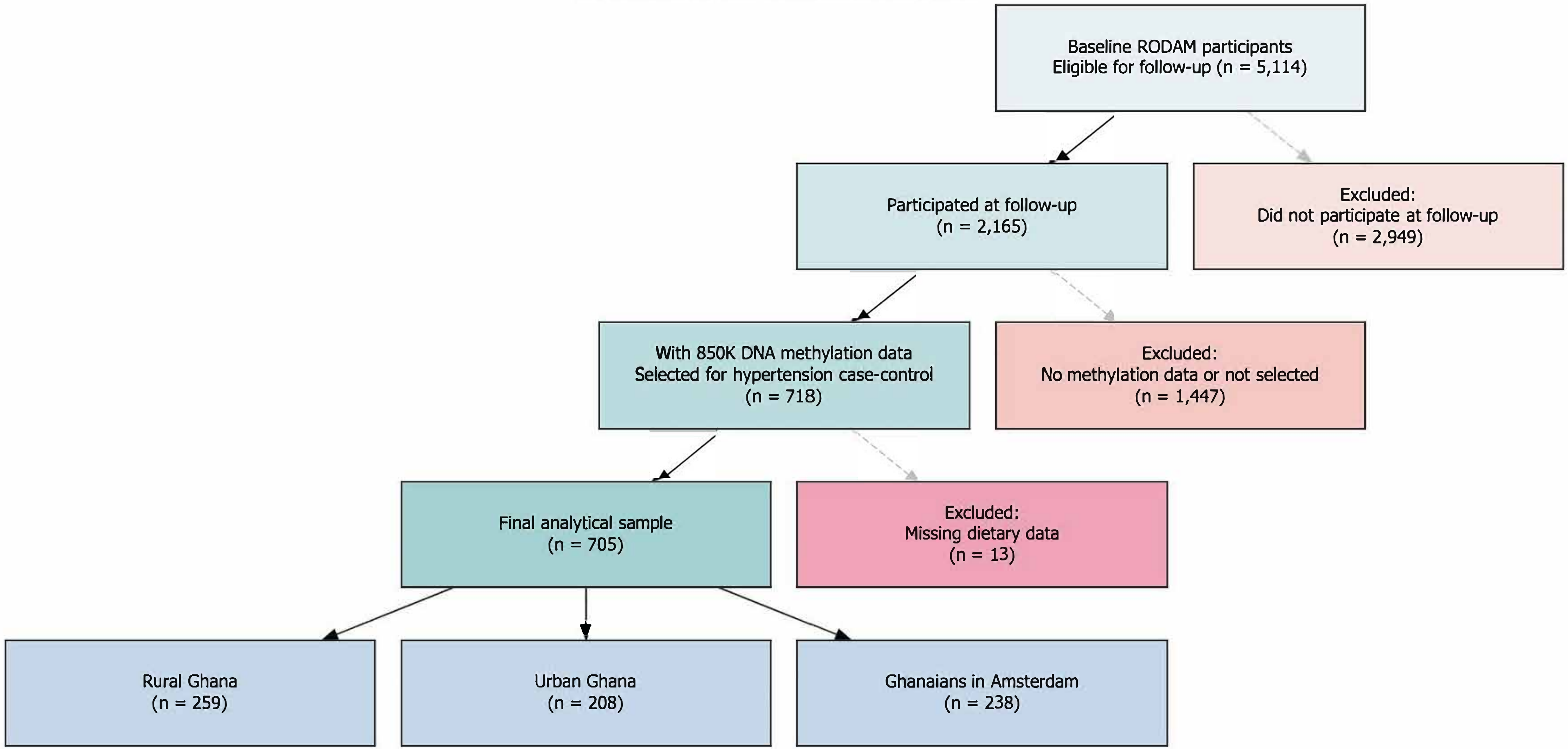

Additional file 1: Table 3 . Estimated blood immune cell proportions by study site

Cells	Rural Ghana (n=259)	Urban Ghana (n=208)	Amsterdam (n=238)	P value
	Mean(SD)	Mean (SD)	Mean (SD)	
Bcell	0.094 (0.044)	0.084 (0.035)	0.079 (0.034)	<0.001
CD4T	0.199 (0.073)	0.195 (0.067)	0.196 (0.071)	0.789
CD8T	4.195 (4.777)	2.741 (4.005)	2.778 (3.745)	<0.001
Gran	0.428 (0.107)	0.473 (0.093)	0.487 (0.101)	<0.001
Mono	0.063 (0.035)	0.059 (0.027)	0.064 (0.028)	0.217
NK	0.091 (0.073)	0.083 (0.051)	0.081 (0.057)	0.139

Immune cell proportions were obtained as output from the UCLA online clock calculation platform (Clock Foundation; https://clockfoundation.org/) These proportions were estimated using the Houseman method. Values are presented as mean (SD). P values were calculated using one-way ANOVA to test for differences in estimated blood cell proportions across study sites.

Additional file 1: Table 4. Association between specific food groups and epigenetic age acceleration, **adjusted for cell-type composition** (Full adjusted)

Specific food groups	Horvath EAA β (95%CI)	Hannum EAA β (95%CI)	GrimAge EAA β (95%CI)	PhenoAge EAA β (95%CI)
**Rural Ghana (n=259)**				
Alcoholic_beverages	0 (-0.004–0.003)	0.003 (-0.001–0.006)	0.001 (-0.001–0.003)	0.001 (-0.004–0.005)
Cakes_sweets	-0.026 (-0.055–0.004)	-0.025 (-0.052–0.002)	-0.009 (-0.026–0.007)	**-0.042 (-0.078–-0.006) ***
Coffee_tea	0 (-0.002–0.002)	0 (-0.002–0.002)	0 (-0.001–0.001)	0 (-0.002–0.002)
Condiments	-0.01 (-0.029–0.01)	-0.016 (-0.034–0.002)	-0.01 (-0.021–0.001)	-0.02 (-0.044–0.004)
Cooking_fats	0.128 (-8.046–8.301)	-2.468 (-9.942–5.005)	-0.92 (-5.5–3.661)	-3.377 (-13.321–6.567)
Dairy_products	-0.008 (-0.024–0.008)	-0.009 (-0.023–0.006)	0.001 (-0.007–0.01)	0.003 (-0.017–0.022)
Egg	0.008 (-0.056–0.073)	0.007 (-0.052–0.066)	0.012 (-0.024–0.048)	0.039 (-0.039–0.118)
Fermente_maize_products	0 (-0.004–0.005)	0.002 (-0.002–0.006)	0 (-0.002–0.003)	0 (-0.005–0.006)
Fish	**-0.028 (-0.056–-0.001) ***	-0.014 (-0.04–0.011)	-0.007 (-0.023–0.009)	**-0.026 (-0.062– -0.006) ***
Fruit	0 (-0.004–0.003)	0 (-0.003–0.003)	0 (-0.002–0.002)	-0.001 (-0.006–0.003)
Legumes	-0.01 (-0.024–0.005)	0.006 (-0.008–0.019)	0.003 (-0.006–0.011)	-0.001 (-0.019–0.017)
Margarine	-0.169 (-0.416–0.078)	-0.123 (-0.35–0.103)	-0.098 (-0.236–0.041)	-0.017 (-0.318–0.285)
Meaty_mixed_dishes	-0.004 (-0.019–0.011)	0 (-0.014–0.013)	-0.004 (-0.012–0.004)	-0.002 (-0.02–0.016)
Nuts_seeds	0.033 (-0.038–0.104)	-0.009 (-0.074–0.056)	-0.012 (-0.052–0.028)	0 (-0.087–0.086)
Olive_oil	-0.1 (-4.708–4.508)	-1.914 (-6.124–2.296)	0.763 (-1.819–3.344)	1.8 (-3.807–7.407)
Other_oils	0.022 (-0.041–0.085)	0.008 (-0.05–0.065)	-0.007 (-0.042–0.028)	0.017 (-0.059–0.094)

Specific food groups	Horvath EAA β (95%CI)	Hannum EAA β (95%CI)	GrimAge EAA β (95%CI)	PhenoAge EAA β (95%CI)
Palm_oil	0.147 (-0.181–0.475)	0.059 (-0.241–0.36)	-0.065 (-0.249–0.119)	0.13 (-0.27–0.529)
Potatoes	0.007 (-0.022–0.036)	-0.007 (-0.034–0.02)	0 (-0.017–0.016)	0 (-0.036–0.035)
Poultry	0.011 (-0.03–0.051)	-0.013 (-0.05–0.024)	-0.005 (-0.028–0.017)	**0.046 (-0.003–0.095)** *
Processed_meat	-0.005 (-0.056–0.047)	-0.017 (-0.064–0.03)	**-0.031 (-0.059–-0.002)** *	0.013 (-0.049–0.075)
Red_meat	-0.009 (-0.029–0.012)	-0.009 (-0.028–0.01)	-0.002 (-0.013–0.01)	-0.006 (-0.032–0.019)
Rice_pasta	-0.002 (-0.01–0.006)	0 (-0.008–0.007)	0 (-0.004–0.005)	-0.001 (-0.011–0.009)
Roots_tubers_plantain	0.002 (0–0.004)	0.001 (-0.001–0.003)	0 (-0.001–0.001)	**0.003 (0.001–0.005)** *
Sodas_juices	-0.002 (-0.006–0.002)	-0.002 (-0.006–0.001)	0.001 (-0.001–0.003)	-0.001 (-0.005–0.004)
Sweet_spreads	-0.165 (-2.09–1.761)	-0.274 (-2.036–1.487)	0.076 (-1.004–1.155)	1.323 (-1.016–3.662)
Vegetable_soups_stews_sauces	-0.001 (-0.004–0.001)	-0.001 (-0.003–0.002)	0.001 (-0.001–0.002)	-0.001 (-0.004–0.002)
Vegetables	0 (-0.007–0.006)	-0.001 (-0.007–0.005)	0.001 (-0.002–0.005)	-0.001 (-0.009–0.006)
Vegetarian_mixed_dishes	-0.025 (-0.064–0.014)	-0.026 (-0.061–0.01)	-0.011 (-0.033–0.01)	-0.042 (-0.089–0.005)
White_bread_cereals	0 (-0.009–0.008)	0.002 (-0.005–0.01)	0.001 (-0.004–0.005)	-0.002 (-0.012–0.009)
Whole_grain_bread_cereals	-0.001 (-0.016–0.015)	-0.001 (-0.015–0.014)	-0.002 (-0.011–0.007)	0.001 (-0.018–0.02)
**Urban Ghana (n=208)**				
Alcoholic_beverages	0.011 (-0.007–0.029)	0.004 (-0.012–0.019)	0.002 (-0.009–0.012)	0.017 (-0.005–0.038)
Cakes_sweets	-0.019 (-0.066–0.028)	-0.013 (-0.054–0.027)	0.015 (-0.013–0.043)	-0.046 (-0.102–0.01)
Coffee_tea	0 (-0.002–0.002)	0.001 (-0.001–0.002)	0 (-0.002–0.001)	-0.002 (-0.005–0) *
Condiments	-0.005 (-0.024–0.015)	-0.004 (-0.02–0.013)	0.005 (-0.007–0.017)	-0.012 (-0.035–0.012)

Specific food groups	Horvath EAA<br>β (95%CI)	Hannum EAA<br>β (95%CI)	GrimAge EAA<br>β (95%CI)	PhenoAge EAA<br>β (95%CI)
Cooking_fats	-14.204 (-33.273–4.865)	**-15.614 (-31.929–-0.721) ***	-5.47 (-17.046–6.105)	-24.404 (-47.256–1.553)
Dairy_products	-0.004 (-0.014–0.007)	-0.005 (-0.014–0.004)	0.006 (-0.001–0.012)	**-0.018 (-0.03–-0.005) ***
Egg	-0.026 (-0.081–0.029)	-0.024 (-0.072–0.023)	-0.006 (-0.04–0.027)	-0.052 (-0.118–0.015)
Fermente_maize_products	-0.001 (-0.005– 0.002)	**-0.002 (-0.005–-0.001)***	-0.002 (-0.004–0.001)	**-0.006 (-0.01–-0.001) ***
Fish	0.014 (-0.005–0.033)	0.013 (-0.004–0.029)	0.001 (-0.011–0.012)	-0.013 (-0.036–0.01)
Fruit	0.002 (-0.004–0.007)	0.002 (-0.003–0.006)	-0.001 (-0.004–0.003)	-0.001 (-0.007–0.005)
Legumes	-0.006 (-0.024–0.011)	-0.009 (-0.024–0.006)	0.002 (-0.008–0.013)	-0.002 (-0.022–0.019)
Margarine	-0.026 (-0.241–0.19)	-0.095 (-0.279–0.09)	0.113 (-0.016–0.243)	-0.207 (-0.465–0.051)
Meaty_mixed_dishes	0.006 (-0.009–0.022)	0.002 (-0.012–0.015)	0.007 (-0.002–0.017)	0.005 (-0.014–0.024)
Nuts_seeds	0.025 (-0.035–0.085)	0.049 (-0.002–0.1)	**0.042 (0.006–0.078) ***	0.029 (-0.044–0.101)
Olive_oil	0.258 (-0.674–1.191)	-0.11 (-0.911–0.692)	-0.279 (-0.842–0.285)	-0.547 (-1.669–0.575)
Other_oils	**-0.059 (-0.121–0.003) ***	-0.034 (-0.087–0.02)	-0.005 (-0.042–0.033)	-0.003 (-0.078–0.073)
Palm_oil	-0.05 (-0.356–0.255)	0.014 (-0.249–0.276)	0.046 (-0.138–0.231)	-0.185 (-0.553–0.182)
Potatoes	-0.017 (-0.045–0.012)	0.006 (-0.019–0.03)	0.011 (-0.006–0.028)	0.001 (-0.034–0.036)
Poultry	-0.01 (-0.05–0.03)	-0.012 (-0.046–0.023)	0.008 (-0.016–0.032)	-0.017 (-0.065–0.031)
Processed_meat	-0.015 (-0.061–0.032)	0.009 (-0.031–0.049)	**0.03 (0.002–0.058) ***	0.022 (-0.034–0.078)
Red_meat	-0.008 (-0.029–0.013)	**-0.021 (-0.039–-0.003) ***	0.011 (-0.002–0.024)	-0.017 (-0.043–0.008)
Rice_pasta	-0.002 (-0.01–0.007)	-0.003 (-0.01–0.004)	0.004 (-0.001–0.009)	-0.001 (-0.011–0.009)
Roots_tubers_plantain	0 (-0.005–0.006)	0.003 (-0.001–0.008)	0 (-0.003–0.004)	-0.003 (-0.01–0.003)

Specific food groups	Horvath EAA<br>β (95%CI)	Hannum EAA<br>β (95%CI)	GrimAge EAA<br>β (95%CI)	PhenoAge EAA<br>β (95%CI)
Sodas_juices	**0.006 (0.001–0.01)** *	0.001 (-0.003–0.005)	-0.001 (-0.003–0.002)	0.001 (-0.004–0.007)
Sweet_spreads	0.257 (-0.152–0.666)	0.419 (0.072–0.767) *	-0.171 (-0.418–0.077)	0.145 (-0.35–0.639)
Vegetable_soups_stews_sauces	-0.001 (-0.003–0.001)	-0.001 (-0.002–0.001)	-0.001 (-0.002–0)	0.001 (-0.002–0.003)
Vegetables	0 (-0.007–0.007)	0.001 (-0.004–0.007)	0.001 (-0.003–0.005)	-0.005 (-0.013–0.003)
Vegetarian_mixed_dishes	0.004 (-0.037–0.046)	-0.012 (-0.048–0.023)	0.014 (-0.011–0.039)	0.007 (-0.043–0.057)
White_bread_cereals	0 (-0.009–0.008)	0.001 (-0.006–0.008)	0 (-0.005–0.005)	0 (-0.011–0.01)
Whole_grain_bread_cereals	0.003 (-0.009–0.015)	0.001 (-0.01–0.011)	**0.007 (0.001–0.015)** *	0.001 (-0.013–0.016)
**Amsterdam (Ghanaians, n=238)**				
Alcoholic_beverages	-0.001 (-0.004–0.001)	0 (-0.002–0.002)	0.001 (0–0.002)	0.001 (-0.001–0.004)
Cakes_sweets	**-0.048 (-0.084–-0.011)** *	-0.015 (-0.043–0.014)	0.017 (-0.001–0.036)	-0.026 (-0.063–0.012)
Coffee_tea	0 (-0.001–0.001)	0 (0–0.001)	0 (0–0.001)	0 (-0.001–0.001)
Condiments	0.001 (-0.014–0.011)	**0.010 (0.001–0.021)** *	0.001 (-0.006–0.007)	**0.012 (0.001–0.025)** *
Cooking_fats	-1.552 (-4.657–1.553)	-1.253 (-3.661–1.155)	**-2.784 (-4.31–-1.259)** *	-2.421 (-5.544–0.745)
Dairy_products	-0.003 (-0.01–0.005)	0.001 (-0.005–0.006)	0.002 (-0.001–0.006)	0.004 (-0.003–0.011)
Egg	-0.022 (-0.074–0.029)	-0.012 (-0.052–0.028)	-0.009 (-0.035–0.017)	-0.027 (-0.08–0.025)
Fermente_maize_products	-0.004 (-0.015–0.006)	0 (-0.008–0.008)	0.002 (-0.004–0.007)	0 (-0.01–0.01)
Fish	0.01 (-0.011–0.031)	0.003 (-0.014–0.019)	0.006 (-0.004–0.017)	0.013 (-0.008–0.034)
Fruit	0 (-0.003–0.002)	0 (-0.001–0.002)	-0.001 (-0.002–0.001)	0.001 (-0.001–0.004)
Legumes	0.007 (-0.014–0.028)	-0.014 (-0.031–0.002)	0 (-0.01–0.011)	0.004 (-0.017–0.025)

Specific food groups	Horvath EAA<br>β (95%CI)	Hannum EAA<br>β (95%CI)	GrimAge EAA<br>β (95%CI)	PhenoAge EAA<br>β (95%CI)
Margarine	0.057 (-0.076–0.189)	0.038 (-0.065–0.141)	0.035 (-0.032–0.102)	0.004 (-0.131–0.139)
Meaty_mixed_dishes	0.005 (-0.014–0.023)	0.006 (-0.009–0.02)	-0.004 (-0.013–0.005)	0.005 (-0.014–0.024)
Nuts_seeds	0.013 (-0.025–0.052)	0.018 (-0.012–0.048)	0.001 (-0.018–0.021)	0.011 (-0.029–0.05)
Olive_oil	-0.079 (-0.432–0.274)	-0.176 (-0.449–0.096)	-0.117 (-0.294–0.061)	-0.218 (-0.575–0.14)
Other_oils	0 (-0.039–0.038)	0.011 (-0.019–0.041)	0 (-0.019–0.02)	0.014 (-0.026–0.053)
Palm_oil	-1.07 (-2.108–0.032)	-0.674 (-1.482–0.133)	0.322 (-0.205–0.849)	**-1.179 (-2.231–-0.126)** *
Potatoes	-0.002 (-0.017–0.013)	-0.006 (-0.018–0.005)	0.001 (-0.007–0.008)	0.004 (-0.012–0.019)
Poultry	-0.012 (-0.04–0.016)	0.003 (-0.018–0.025)	-0.007 (-0.021–0.007)	0.004 (-0.024–0.033)
Processed_meat	-0.035 (-0.074–0.004)	-0.009 (-0.04–0.021)	0.007 (-0.013–0.027)	**-0.04 (-0.08–0)** *
Red_meat	-0.001 (-0.023–0.021)	0.003 (-0.014–0.021)	-0.002 (-0.013–0.009)	-0.013 (-0.036–0.009)
Rice_pasta	-0.003 (-0.012–0.005)	0.002 (-0.004–0.009)	0 (-0.004–0.004)	0.003 (-0.006–0.011)
Roots_tubers_plantain	-0.001 (-0.007–0.005)	-0.001 (-0.006–0.003)	0.001 (-0.002–0.004)	-0.002 (-0.008–0.004)
Sodas_juices	0 (-0.003–0.002)	0 (-0.002–0.002)	0.001 (-0.001–0.002)	0 (-0.002–0.003)
Sweet_spreads	-0.023 (-0.176–0.13)	-0.043 (-0.161–0.076)	0.035 (-0.042–0.112)	0.011 (-0.144–0.166)
Vegetable_soups_stews_sauces	0.001 (-0.001–0.003)	0.001 (-0.001–0.002)	**0.001 (0–0.002)** *	0.001 (-0.001–0.003)
Vegetables	-0.001 (-0.005–0.004)	0 (-0.003–0.004)	0 (-0.003–0.002)	0 (-0.004–0.005)
Vegetarian_mixed_dishes	0.02 (-0.016–0.057)	0.021 (-0.008–0.049)	0.001 (-0.018–0.019)	**0.047 (0.01–0.084)** *
White_bread_cereals	-0.002 (-0.011–0.007)	-0.001 (-0.008–0.006)	0 (-0.004–0.005)	0.005 (-0.004–0.014)
Whole_grain_bread_cereals	-0.001 (-0.015–0.013)	0.008 (-0.003–0.018)	0.001 (-0.006–0.008)	0.007 (-0.007–0.021)

This table presents the associations between intake of specific food groups (grams/day) and four measures of epigenetic age acceleration (Horvath EAA, Hannum EAA, GrimAge EAA, and PhenoAge EAA), stratified by study site (rural Ghana, urban Ghana, and Amsterdam). **Beta coefficients (β) and 95% confidence intervals (CI)** are derived from linear regression models fully **adjusted** for age, sex, education level, smoking status, physical activity, BMI, alcohol intake, diabetes status, and hypertension status. Positive β values indicate greater epigenetic age acceleration, while negative values suggest slower biological aging relative to chronological age. Asterisks (*) and bolded denote statistically significant associations based on the 95% confidence interval ($p \leq 0.05$). Bolded estimates indicate a consistent direction of association across all three study sites. Overall significance is determined using the "2+ rule": an association is considered robust if at least two clocks show statistically significant associations and all four clocks show a consistent direction of effect.

Additional file 1: Table 5: Fully Adjusted Associations Between Dietary Patterns, Plant-Based and Animal-Based Food Proportions, and Epigenetic Age Acceleration Among Ghanaian Adults, Stratified by Study Site, **adjusted for estimated blood cell proportions.**

Specific food groups	Horvath EAA<br>Adjusted β (95%CI)	Hannum EAA<br>Adjusted β (95%CI)	GrimAge EAA<br>Adjusted β (95%CI)	PhenoAge EAA<br>Adjusted β (95%CI)
**Data driven dietary patterns**				
**Rural Ghana (n=259)**				
DP1 (Plant-based & mixed traditional foods)	0.209 (-0.898–1.317)	0.114 (-0.9–1.128)	0.149 (-0.472–0.77)	0.027 (-1.322–1.377)
DP2 (Animal-based and fried/staple-rich foods)	-0.691 (-1.489–0.106)	**-0.701 (-1.43–0.028)** *	-0.327 (-0.775–0.121)	-0.513 (-1.488–0.462)
DP3 (Modern/processed foods)	-0.045 (-0.806–0.716)	0.342 (-0.353–1.037)	-0.003 (-0.43–0.423)	0.015 (-0.912–0.941)
**Urban Ghana (n=208)**				
DP1 (Plant-based & mixed traditional foods)	0.119 (-1.07–1.307)	0.622 (-0.395–1.639)	0.19 (-0.529–0.908)	-0.684 (-2.113–0.746)
DP2 (Animal-based and fried/staple-rich foods)	-0.205 (-1.072–0.662)	-0.539 (-1.28–0.202)	**0.625 (0.108–1.142)** *	-0.659 (-1.7–0.383)
DP3 (Modern/processed foods)	-0.131 (-1.019–0.757)	-0.14 (-0.902–0.622)	0.172 (-0.365–0.708)	-0.795 (-1.859–0.269)
**Amsterdam (Ghanaians, n=238)**				
DP1 (Plant-based & mixed traditional foods)	0.036 (-0.628–0.7)	0.225 (-0.289–0.739)	0.049 (-0.286–0.384)	0.581 (-0.089–1.251)
DP2 (Animal-based and fried/staple-rich foods)	-0.398 (-1.113–0.317)	0.025 (-0.532–0.581)	0.087 (-0.275–0.449)	-0.31 (-1.037–0.417)
DP3 (Modern/processed foods)	0.188 (-0.852–1.228)	0.19 (-0.617–0.996)	0.188 (-0.336–0.713)	0.949 (-0.1–1.998)
**Proportion of plant/animal-based food intake**				
**Rural Ghana (n=259)**				
Proportion healthful plant-based	0.047 (-0.015–0.109)	0.028 (-0.029–0.085)	-0.002 (-0.037–0.033)	0.038 (-0.038–0.114)

Specific food groups	Horvath EAA<br>Adjusted β (95%CI)	Hannum EAA<br>Adjusted β (95%CI)	GrimAge EAA<br>Adjusted β (95%CI)	PhenoAge EAA<br>Adjusted β (95%CI)
Proportion unhealthful plant-based	-0.009 (-0.081–0.064)	0.016 (-0.05–0.083)	0.005 (-0.036–0.046)	-0.004 (-0.092–0.085)
Proportion animal-based	-0.028 (-0.087–0.03)	-0.024 (-0.078–0.03)	0.006 (-0.027–0.039)	-0.017 (-0.089–0.055)
**Urban Ghana (n=208)**				
Proportion healthful plant-based	0.036 (-0.042–0.114)	**0.062 (-0.005–0.128) ***	0.034 (-0.013–0.08)	0.02 (-0.074–0.114)
Proportion unhealthful plant-based	0.005 (-0.075–0.086)	-0.027 (-0.096–0.041)	-0.017 (-0.065–0.032)	-0.062 (-0.158–0.034)
Proportion animal-based	-0.023 (-0.083–0.037)	-0.021 (-0.072–0.031)	-0.014 (-0.051–0.022)	0.028 (-0.045–0.101)
**Amsterdam (Ghanaians, n=238)**				
Proportion healthful plant-based	-0.002 (-0.061–0.057)	-0.01 (-0.056–0.036)	**-0.031 (-0.061–-0.002) ***	-0.021 (-0.081–0.039)
Proportion unhealthful plant-based	-0.032 (-0.108–0.043)	-0.005 (-0.064–0.054)	0.006 (-0.032–0.045)	-0.003 (-0.08–0.074)
Proportion animal-based	0.024 (-0.031–0.08)	0.006 (-0.037–0.049)	**0.026 (-0.001–0.054) ***	0.019 (-0.037–0.075)

This table presents the associations between data-driven dietary patterns, proportions of plant-based and animal-based food intake, and four measures of epigenetic age acceleration (Horvath EAA, Hannum EAA, GrimAge EAA, and PhenoAge EAA), stratified by study site (rural Ghana, urban Ghana, and Amsterdam). The values shown are beta coefficients (β) and 95% confidence intervals (CI), derived from linear regression models. Associations are shown after full adjustment (in brackets) for age, sex, education level, smoking status, physical activity, BMI, alcohol intake, diabetes status, and hypertension status. Positive β values indicate greater epigenetic age acceleration, while negative values reflect slower biological aging relative to chronological age. Asterisks (*) and bolded indicate statistically significant associations based on the 95% confidence interval. Overall significance is determined using the "2+ rule," whereby an association is considered robust if at least two epigenetic clocks show statistically significant associations and all four clocks show a consistent direction of effect.

Additional file 1: Table 6. Stepwise models for statistically significant findings in both main and sensitivity analyses.

Food	Clock	Model 1: Crude	Model 2: Age + Sex	Model 3: Lifestyle +BMI	Model 4: Comorbidity	Model 5: + Cells
**Rural Ghana**						
Fish	Horvath EAA	-0.016 (-0.046–0.013)	-0.033 (-0.062–-0.003) *	-0.034 (-0.065–-0.003) *	-0.035 (-0.066–-0.004) *	**-0.028 (-0.056–-0.001) ***
	Hannum EAA	-0.022 (-0.052–0.008)	-0.029 (-0.059–0.002)	-0.03 (-0.061–0.001) *	-0.027 (-0.059–0.004)	**-0.014** (-0.04–0.011)
	GrimAge EAA	-0.004 (-0.022–0.014)	-0.011 (-0.028–0.007)	-0.012 (-0.027–0.008)	-0.008 (-0.025–0.009)	**-0.007** (-0.023–0.009)
	PhenoAge EAA	-0.032 (-0.069–0.005)	-0.039 (-0.077–-0.001) *	-0.041 (-0.08–-0.002) *	-0.039 (-0.078–-0.003) *	**-0.028 (-0.062–-0.006)***
**Urban Ghana**						
Fermented maize products	Horvath EAA	-0.002 (-0.006–0.001)	-0.002 (-0.006–0.001)	-0.003 (-0.006–0.001)	-0.002 (-0.006–0.001)	**-0.001** (-0.005–0.002)
	Hannum EAA	-0.004 (-0.007–-0.001) *	-0.004 (-0.007–-0.001) *	-0.004 (-0.008–-0.001) *	-0.004 (-0.007–-0.002) *	**-0.002 (-0.005–-0.001)***
	GrimAge EAA	-0.002 (-0.004–0.001)	-0.002 (-0.004–0.001)	-0.002 (-0.005–-0.001) *	-0.002 (-0.004–0.001)	**-0.002** (-0.004–0.001)
	PhenoAge EAA	-0.008 (-0.012–-0.003) *	-0.008 (-0.012–-0.003) *	-0.008 (-0.013–-0.004) *	-0.007 (-0.012–-0.003) *	**-0.006 (-0.01–-0.001) ***
**Amsterdam (Ghanaians)**						
Condiments	Horvath EAA	0.003 (-0.016–0.009)	0.002 (-0.015–0.01)	0.001 (-0.014–0.011)	0.001 (-0.013–0.012)	**0.001** (-0.014–0.011)
	Hannum EAA	0.01 (-0.002–0.021)	0.01 (-0.001–0.021)	0.011 (0–0.022) *	0.011 (0.001–0.023) *	**0.010 (0.001–0.021) ***
	GrimAge EAA	0.002 (-0.005–0.009)	0.003 (-0.004–0.01)	0.003 (-0.004–0.01)	0.003 (-0.004–0.011)	**0.001** (-0.006–0.007)
	PhenoAge EAA	0.013 (0.001–0.027) *	0.013 (0–0.027) *	0.015 (0.001–0.029) *	0.016 (0.002–0.03) *	**0.012 (0.001–0.025) ***

Values are beta (95% CI). Asterisk (*) indicates statistically significant confidence intervals. Rows represent epigenetic clocks; columns represent sequential adjustment models. Bold and aasterix is robust statistically significant findings.

Model definitions:

Model 1 (**Crude**) includes no covariates.

Model 2 (**Age + Sex**) is adjusted for chronological age and sex.

Model 3 (**Lifestyle and anthropometry**) is additionally adjusted for education level, smoking status, physical activity, alcohol intake, and body mass index (BMI).

Model 4 (**Comorbidity**) is additionally adjusted for diabetes and hypertension.

Model 5 (**+ Cells**) is additionally adjusted for estimated blood cell proportions ($CD4^+$ T cells, $CD8^+$ T cells, natural killer cells, B cells, monocytes, and granulocytes).

Additional file 1: Table 7a: Association between specific food groups and epigenetic age acceleration (Full adjusted primary models, **using willet cut offs**)

**Specific food groups**	**Horvath EAA β (95%CI)**	**Hannum EAA β (95%CI)**	**GrimAge EAA β (95%CI)**	**PhenoAge EAA β (95%CI)**
**Rural Ghana (n=227)**				
Alcoholic_beverages	-0.022 (-0.037–0.007)	-0.016 (0.032–0.001)	-0.002 (-0.01–0.007)	-0.022 (-0.0410–.003)
Cakes_sweets	-0.033 (-0.073–0.008)	-0.044 (-0.086–0.003)	-0.012 (-0.035–0.011)	**-0.062 (-0.113–-0.011) ***
Coffee_tea	0.001 (-0.001–0.004)	0 (-0.003–0.003)	0 (-0.002–0.001)	0 (-0.003–0.004)
Condiments	-0.011 (-0.036–0.014)	-0.02 (-0.046–0.005)	-0.01 (-0.024–0.004)	-0.02 (-0.052–0.011)
Cooking_fats	0.003 (-11.078–11.083)	-5.534 (-16.818–5.751)	-2.585 (-8.85–3.68)	-12.722 (-26.552–1.108)
Dairy_products	-0.016 (-0.038–0.005)	**-0.024 (-0.046–-0.002) ***	0.002 (-0.01–0.015)	-0.015 (-0.042–0.013)
Egg	-0.006 (-0.083–0.071)	-0.016 (-0.095–0.062)	0.013 (-0.03–0.057)	0.021 (-0.075–0.118)
Fermente_maize_products	-0.002 (-0.008–0.004)	0.001 (-0.005–0.006)	0.001 (-0.002–0.004)	-0.001 (-0.008–0.006)
Fish	**-0.034** (-0.068–0.001)	**-0.031 (-0.067–-0.004) ***	**-0.006** (-0.025–0.014)	**-0.042 (-0.085–-0.001) ***
Fruit	0.001 (-0.004–0.006)	0.002 (-0.003–0.008)	0.001 (-0.002–0.004)	-0.001 (-0.008–0.005)
Legumes	-0.013 (-0.031–0.005)	-0.002 (-0.02–0.017)	0.005 (-0.005–0.015)	-0.005 (-0.028–0.018)
Margarine	-0.156 (-0.46–0.149)	-0.157 (-0.467–0.154)	-0.124 (-0.296–0.048)	-0.064 (-0.448–0.32)
Meaty_mixed_dishes	-0.005 (-0.022–0.013)	-0.002 (-0.019–0.016)	-0.004 (-0.014–0.006)	0 (-0.022–0.022)
Nuts_seeds	-0.007 (-0.098–0.084)	-0.048 (-0.141–0.045)	-0.006 (-0.057–0.046)	-0.024 (-0.138–0.09)
Olive_oil	-1.421 (-6.612–3.77)	-2.79 (-8.079–2.498)	0.757 (-2.183–3.697)	0.542 (-5.992–7.076)

Specific food groups	Horvath EAA<br>β (95%CI)	Hannum EAA<br>β (95%CI)	GrimAge EAA<br>β (95%CI)	PhenoAge EAA<br>β (95%CI)
Other_oils	-0.051 (-0.147–0.044)	-0.062 (-0.159–0.035)	-0.038 (-0.092–0.016)	-0.099 (-0.218–0.021)
Palm_oil	0.227 (-0.196–0.65)	0.141 (-0.291–0.573)	-0.048 (-0.288–0.192)	0.24 (-0.292–0.772)
Potatoes	0.017 (-0.026–0.059)	0.002 (-0.042–0.046)	0.002 (-0.023–0.026)	0.02 (-0.034–0.073)
Poultry	0.026 (-0.024–0.075)	-0.02 (-0.07–0.03)	-0.001 (-0.029–0.027)	0.055 (-0.006–0.117)
Processed_meat	0.021 (-0.08–0.122)	-0.022 (-0.125–0.081)	-0.041 (-0.098–0.016)	0.018 (-0.109–0.145)
Red_meat	-0.017 (-0.044–0.011)	**-0.03 (-0.058–-0.003)***	-0.001 (-0.017–0.014)	-0.017 (-0.051–0.017)
Rice_pasta	-0.003 (-0.014–0.007)	-0.001 (-0.011–0.01)	0 (-0.006–0.006)	0 (-0.013–0.012)
Roots_tubers_plantain	0 (-0.003–0.004)	-0.001 (-0.005–0.002)	0 (-0.002–0.001)	-0.001 (-0.005–0.003)
Sodas_juices	-0.001 (-0.006–0.003)	-0.001 (-0.006–0.003)	0.001 (-0.002–0.003)	0 (-0.005–0.006)
Sweet_spreads	-0.471 (-3.054–2.112)	1.048 (-1.585–3.682)	0.311 (-1.152–1.773)	2.219 (-1.017–5.455)
Vegetable_soups_stews_sauces	0 (-0.003–0.003)	0 (-0.003–0.003)	0.001 (-0.001–0.003)	0.001 (-0.003–0.005)
Vegetables	0 (-0.008–0.008)	-0.002 (-0.01–0.006)	0.002 (-0.003–0.006)	-0.003 (-0.013–0.007)
Vegetarian_mixed_dishes	-0.016 (-0.063–0.032)	-0.006 (-0.054–0.043)	-0.005 (-0.032–0.022)	-0.023 (-0.082–0.037)
White_bread_cereals	-0.002 (-0.013–0.009)	0.005 (-0.006–0.017)	0 (-0.006–0.007)	-0.004 (-0.018–0.01)
Whole_grain_bread_cereals	0.002 (-0.024–0.028)	0.002 (-0.024–0.028)	0 (-0.014–0.015)	0.006 (-0.026–0.038)
**Urban Ghana (n=202)**				
Alcoholic_beverages	0.013 (-0.005–0.031)	0.01 (-0.007–0.027)	0.007 (-0.004–0.019)	0.028 (0.005–0.052)
Cakes_sweets	0.006 (-0.042–0.055)	0.005 (-0.04–0.05)	0.014 (-0.017–0.046)	**0.071 (0.008–0.132) ***
Coffee_tea	-0.033 (-0.08–0.014)	-0.027 (-0.071–0.016)	0.011 (-0.02–0.041)	**-0.064 (-0.123–-0.005) ***

Specific food groups	Horvath EAA<br>β (95%CI)	Hannum EAA<br>β (95%CI)	GrimAge EAA<br>β (95%CI)	PhenoAge EAA<br>β (95%CI)
Condiments	0 (-0.002–0.002)	0.001 (-0.001–0.003)	0 (-0.002–0.001)	-0.002 (-0.005–0.001)
Cooking_fats	0.002 (-0.018–0.022)	0.003 (-0.015–0.021)	0.005 (-0.007–0.018)	-0.007 (-0.032–0.018)
Dairy_products	-14.45 (-33.387–4.487)	-13.529 (-31.039–3.981)	-3.588 (-15.776–8.601)	-18.774 (-42.817–5.269)
Egg	0 (-0.011–0.011)	-0.002 (-0.012–0.008)	0.005 (-0.002–0.012)	-0.011 (-0.024–0.003)
Fermente_maize_products	-0.052 (-0.107–0.002)	**-0.064 (-0.114–-0.014) ***	-0.008 (-0.043–0.028)	**-0.074 (-0.143–-0.004) ***
Fish	-0.002 (-0.006–0.002)	-0.003 (-0.007–0.001)	**-0.003 (-0.006–-0.001)**	**-0.006 (-0.011–0.001) ***
Fruit	0.009 (-0.01–0.028)	0.004 (-0.014–0.022)	-0.002 (-0.015–0.01)	-0.022 (-0.046–0.003)
Legumes	0.003 (-0.003–0.008)	0.002 (-0.003–0.007)	-0.002 (-0.006–0.001)	-0.001 (-0.008–0.006)
Margarine	0.002 (-0.017–0.02)	-0.005 (-0.022–0.012)	0.002 (-0.01–0.013)	0.01 (-0.013–0.034)
Meaty_mixed_dishes	-0.015 (-0.233–0.203)	-0.059 (-0.261–0.142)	0.131 (-0.007–0.269)	-0.166 (-0.442–0.11)
Nuts_seeds	0.009 (-0.007–0.025)	0.004 (-0.011–0.019)	0.008 (-0.003–0.018)	0.009 (-0.012–0.029)
Olive_oil	0.044 (-0.023–0.11)	0.062 (-0.001–0.123)	0.044 (-0.002–0.086)	0.062 (-0.022–0.146)
Other_oils	0.21 (-0.722–1.141)	-0.174 (-1.035–0.688)	-0.248 (-0.844–0.348)	-0.436 (-1.618–0.745)
Palm_oil	**-0.064 (-0.126–-0.002)***	-0.037 (-0.095–0.02)	-0.006 (-0.046–0.034)	-0.01 (-0.089–0.07)
Potatoes	-0.111 (-0.441–0.219)	-0.063 (-0.369–0.242)	0.044 (-0.168–0.256)	-0.12 (-0.54–0.299)
Poultry	-0.016 (-0.044–0.013)	0.004 (-0.023–0.031)	0.009 (-0.009–0.027)	-0.002 (-0.039–0.034)
Processed_meat	0.005 (-0.034–0.045)	0.008 (-0.028–0.045)	0.014 (-0.012–0.039)	0.002 (-0.049–0.052)
Red_meat	-0.017 (-0.064–0.029)	0.01 (-0.034–0.053)	**0.031 (0.001–0.061) ***	0.013 (-0.046–0.072)
Rice_pasta	-0.008 (-0.03–0.014)	**-0.022 (-0.04–0.001) ***	0.013 (0–0.027)	-0.01 (-0.037–0.018)

Specific food groups	Horvath EAA<br>β (95%CI)	Hannum EAA<br>β (95%CI)	GrimAge EAA<br>β (95%CI)	PhenoAge EAA<br>β (95%CI)
Roots_tubers_plantain	0 (-0.008–0.009)	-0.003 (-0.01–0.005)	0.002 (-0.003–0.007)	-0.002 (-0.013–0.008)
Sodas_juices	0.002 (-0.004–0.008)	**0.006 (0.003–0.012) ***	0.001 (-0.003–0.005)	0 (-0.008–0.008)
Sweet_spreads	**0.005 (0.001–0.001)***	0 (-0.004–0.004)	-0.001 (-0.004–0.002)	0 (-0.005–0.006)
Vegetable_soups_stews_sauces	0.228 (-0.175–0.632)	0.286 (-0.086–0.658)	-0.208 (-0.466–0.049)	-0.029 (-0.543–0.485)
Vegetables	0 (-0.002–0.001)	0 (-0.002–0.001)	-0.001 (-0.002–0)	0.002 (-0.001–0.004)
Vegetarian_mixed_dishes	0 (-0.007–0.007)	-0.001 (-0.007–0.006)	-0.001 (-0.005–0.004)	-0.004 (-0.013–0.005)
White_bread_cereals	0.008 (-0.034–0.05)	-0.014 (-0.053–0.025)	0.006 (-0.021–0.033)	0.011 (-0.043–0.064)
Whole_grain_bread_cereals	0.002 (-0.006–0.011)	0.003 (-0.005–0.011)	0 (-0.006–0.005)	0.003 (-0.008–0.014)
**Amsterdam (Ghanaians, n=201)**				
Alcoholic_beverages	-0.002 (-0.006–0.001)	0 (-0.003–0.003)	0.001 (-0.001–0.002)	-0.001 (-0.005–0.003)
Cakes_sweets	-0.065 (-0.113–0.016)	-0.027 (-0.07–0.016)	0.001 (-0.024–0.026)	**-0.059 (-0.111–-0.008) ***
Coffee_tea	0 (-0.002–0.001)	0.000 (-0.001–0.001)	0.000 (-0.001–0.001)	0.001 (-0.002–0.001)
Condiments	0.001 (-0.014–0.016)	0.007 (-0.006–0.02)	-0.004 (-0.012–0.003)	0.011 (-0.005–0.026)
Cooking_fats	-1.883 (-5.187–1.422)	-0.912 (-3.794–1.971)	**-2.061 (-3.702–-0.42) ***	-1.281 (-4.756–2.193)
Dairy_products	-0.005 (-0.014–0.004)	-0.005 (-0.013–0.003)	0 (-0.004–0.005)	0 (-0.01–0.009)
Egg	-0.064 (-0.129–0.002)	-0.038 (-0.095–0.019)	-0.01 (-0.044–0.023)	-0.056 (-0.124–0.013)
Fermente_maize_products	-0.003 (-0.015–0.009)	0.002 (-0.008–0.013)	0.001 (-0.005–0.007)	0.003 (-0.01–0.015)
Fish	0.009 (-0.015–0.034)	-0.001 (-0.022–0.02)	0 (-0.012–0.013)	0.008 (-0.018–0.033)
Fruit	-0.001 (-0.003–0.002)	-0.001 (-0.004–0.002)	-0.001 (-0.002–0)	0 (-0.003–0.003)

Specific food groups	Horvath EAA<br>β (95%CI)	Hannum EAA<br>β (95%CI)	GrimAge EAA<br>β (95%CI)	PhenoAge EAA<br>β (95%CI)
Legumes	-0.011 (-0.037–0.015)	**-0.033 (-0.055–-0.011) ***	-0.001 (-0.014–0.012)	-0.005 (-0.032–0.023)
Margarine	-0.029 (-0.204–0.147)	-0.069 (-0.221–0.084)	-0.005 (-0.093–0.084)	-0.115 (-0.298–0.069)
Meaty_mixed_dishes	0.01 (-0.012–0.031)	0.008 (-0.01–0.027)	-0.008 (-0.019–0.002)	0.002 (-0.02–0.024)
Nuts_seeds	-0.001 (-0.049–0.047)	0.005 (-0.037–0.047)	-0.001 (-0.025–0.023)	0.004 (-0.046–0.055)
Olive_oil	-0.048 (-0.461–0.364)	-0.027 (-0.386–0.332)	-0.11 (-0.316–0.097)	-0.184 (-0.616–0.248)
Other_oils	-0.019 (-0.07–0.032)	-0.002 (-0.046–0.042)	-0.009 (-0.034–0.017)	-0.004 (-0.057–0.049)
Palm_oil	-0.734 (-1.904–0.435)	0.009 (-1.012–1.031)	**0.591 (0.007–1.175) ***	-0.492 (-1.722–0.737)
Potatoes	-0.007 (-0.026–0.012)	-0.011 (-0.027–0.005)	0.002 (-0.007–0.012)	0.004 (-0.016–0.024)
Poultry	-0.005 (-0.038–0.027)	0.006 (-0.023–0.034)	-0.016 (-0.032–0)	0.009 (-0.025–0.043)
Processed_meat	-0.035 (-0.082–0.011)	-0.003 (-0.043–0.038)	0.005 (-0.019–0.028)	-0.041 (-0.09–0.008)
Red_meat	0.011 (-0.016–0.038)	0.019 (-0.004–0.042)	-0.007 (-0.02–0.007)	-0.012 (-0.04–0.016)
Rice_pasta	-0.004 (-0.013–0.005)	-0.001 (-0.01–0.007)	-0.002 (-0.007–0.002)	-0.001 (-0.011–0.008)
Roots_tubers_plantain	-0.001 (-0.008–0.005)	0 (-0.006–0.006)	0 (-0.004–0.003)	-0.004 (-0.011–0.003)
Sodas_juices	-0.001 (-0.005–0.002)	-0.001 (-0.004–0.002)	0 (-0.002–0.002)	-0.002 (-0.005–0.002)
Sweet_spreads	-0.068 (-0.246–0.111)	-0.065 (-0.22–0.09)	0.054 (-0.036–0.144)	0.077 (-0.11–0.264)
Vegetable_soups_stews_sauces	0 (-0.002–0.003)	-0.001 (-0.003–0.001)	**0.001 (0.001–0.003) ***	0.001 (-0.002–0.003)
Vegetables	0 (-0.006–0.005)	-0.002 (-0.007–0.002)	0 (-0.003–0.002)	0 (-0.005–0.005)
Vegetarian_mixed_dishes	0.029 (-0.015–0.073)	0.026 (-0.012–0.064)	-0.008 (-0.031–0.014)	0.018 (-0.028–0.064)
White_bread_cereals	-0.005 (-0.016–0.006)	-0.005 (-0.015–0.004)	0 (-0.005–0.006)	0.002 (-0.009–0.014)

Specific food groups	Horvath EAA<br>β (95%CI)	Hannum EAA<br>β (95%CI)	GrimAge EAA<br>β (95%CI)	PhenoAge EAA<br>β (95%CI)
Whole_grain_bread_cereals	-0.008 (-0.025–0.009)	0.004 (-0.011–0.019)	0.001 (-0.008–0.009)	0 (-0.018–0.018)

This table presents the associations between intake of specific food groups (grams/day) and four measures of epigenetic age acceleration (Horvath EAA, Hannum EAA, GrimAge EAA, and PhenoAge EAA), stratified by study site (rural Ghana, urban Ghana, and Amsterdam). **Beta coefficients (β) and 95% confidence intervals (CI)** are derived from linear regression models fully **adjusted** for age, sex, education level, smoking status, physical activity, BMI, alcohol intake, diabetes status, and hypertension status. Positive β values indicate greater epigenetic age acceleration, while negative values suggest slower biological aging relative to chronological age. Asterisks (*) denote statistically significant associations based on the 95% confidence interval. Bolded estimates indicate a consistent direction of association across all three study sites. Overall significance is determined using the "2+ rule": an association is considered robust if at least two clocks show statistically significant associations and all four clocks show a consistent direction of effect. Analyses were restricted to participants with plausible dietary intake based on Willett cut-offs (500–3500 kcal/day for women and 800–4200 kcal/day for men).

Additional file 1: Table 7b: Fully Adjusted Associations Between Dietary Patterns, Plant-Based and Animal-Based Food Proportions, and Epigenetic Age Acceleration Among Ghanaian Adults, Stratified by Study Site (**used willet cut-offs**).

**Specific food groups**	**Horvath EAA**<br>**Adjusted β (95%CI)**	**Hannum EAA**<br>**Adjusted β (95%CI)**	**GrimAge EAA**<br>**Adjusted β (95%CI)**	**PhenoAge EAA**<br>**Adjusted β (95%CI)**
**Data driven dietary patterns**				
**Rural Ghana (n=227)**				
DP1 (Plant-based & mixed traditional foods)	0.142 (-1.481–1.76)	0.397 (-1.257–2.05)	0.303 (-0.613–1.22)	-0.129 (-2.167–1.91)
DP2 (Animal-based and fried/staple-rich foods)	-0.935 (-2.092–0.222)	**-0.577 (-0.746–-0.408) ***	-0.358 (-1.015–0.299)	-0.853 (-2.312–0.607)
DP3 (Modern/processed foods)	-0.536 (-1.547–0.475)	0.032 (-1.002–1.067)	0.115 (-0.459–0.689)	-0.479 (-1.752–0.794)
**Urban Ghana (n=202)**				
DP1 (Plant-based & mixed traditional foods)	0.502 (-0.767–1.771)	0.734 (-0.437–1.904)	-0.03 (-0.844–0.784)	-0.275 (-1.889–1.339)
DP2 (Animal-based and fried/staple-rich foods)	-0.262 (-1.135–0.615)	-0.567 (-1.373–0.238)	**0.636 (0.082–1.189) ***	-0.699 (-1.807–0.408)
DP3 (Modern/processed foods)	0.153 (-0.896–1.202)	-0.314 (-1.284–0.655)	-0.135 (-0.807–0.536)	-0.399 (-1.731–0.932)
**Amsterdam (Ghanaians, n=201)**				
DP1 (Plant-based & mixed traditional foods)	-0.381 (-1.212–0.453)	-0.461 (-1.184–0.262)	-0.06 (-0.48–0.359)	0.29 (-0.585–1.164)
DP2 (Animal-based and fried/staple-rich foods)	-0.262 (-1.135–0.61)	0.169 (-0.591–0.928)	-0.228 (-0.666–0.209)	-0.547 (-1.46–0.366)
DP3 (Modern/processed foods)	0.037 (-1.185–1.259)	-0.269 (-1.332–0.793)	0.071 (-0.544–0.685)	0.294 (-0.988–1.575)
**Proportion of plant/animal-based food intake**				
**Rural Ghana (n=227)**				

Specific food groups	Horvath EAA<br>Adjusted β (95%CI)	Hannum EAA<br>Adjusted β (95%CI)	GrimAge EAA<br>Adjusted β (95%CI)	PhenoAge EAA<br>Adjusted β (95%CI)
Proportion healthful plant-based	0.038 (–0.19 – 0.665)	0.020 (–0.014 – 0.053)	**0.057 (0.018 – 0.097) ***	0.017 (–0.117 – 0.052)
Proportion unhealthful plant-based	0.013 (–0.222 – 0.047)	**0.053 (0.012 – 0.093) ***	**0.073 (0.029 – 0.096) ***	0.070 (–0.073 – 0.113)
Proportion animal-based	0.025 (–0.211 – 0.661)	0.037 (–0.005 – 0.079)	**0.081 (0.037 – 0.126) ***	0.082 (–0.063 – 0.127)
**Urban Ghana (n=202)**				
Proportion healthful plant-based	0.118 (–0.287 – 0.524)	0.054 (–0.316 – 0.425)	–0.130 (–0.386 – 0.127)	0.238 (–0.272 – 0.749)
Proportion unhealthful plant-based	0.084 (–0.324 – 0.493)	–0.054 (–0.428 – 0.319)	–0.195 (–0.454 – 0.064)	0.143 (–0.372 – 0.657)
Proportion animal-based	0.079 (–0.315 – 0.472)	–0.011 (–0.371 – 0.348)	–0.155 (–0.404 – 0.094)	0.234 (–0.261 – 0.730)
**Amsterdam (Ghanaians, n=201)**				
Proportion healthful plant-based	0.050 (–0.283 – 0.382)	–0.240 (–0.519 – 0.039)	0.106 (–0.060 – 0.271)	–0.074 (–0.423 – 0.275)
Proportion unhealthful plant-based	0.013 (–0.342 – 0.368)	–0.264 (–0.563 – 0.035)	0.131 (–0.046 – 0.308)	–0.079 (–0.452 – 0.293)
Proportion animal-based	0.054 (–0.273 – 0.382)	–0.237 (–0.513 – 0.038)	0.134 (–0.029 – 0.298)	–0.066 (–0.410 – 0.278)

This table presents the associations between data-driven dietary patterns, proportions of plant-based and animal-based food intake, and four measures of epigenetic age acceleration (Horvath EAA, Hannum EAA, GrimAge EAA, and PhenoAge EAA), stratified by study site (rural Ghana, urban Ghana, and Amsterdam). The values shown are beta coefficients (β) and 95% confidence intervals (CI), derived from linear regression models. Associations are shown after full adjustment (in brackets) for age, sex, education level, smoking status, physical activity, BMI, alcohol intake, diabetes status, and hypertension status. Positive β values indicate greater epigenetic age acceleration, while negative values reflect slower biological aging relative to chronological age. Asterisks (*) indicate statistically significant associations based on the 95% confidence interval. Bolded estimates indicate a consistent direction of association across all three study sites. Overall significance is determined using the "2+ rule," whereby an association is considered robust if at least two epigenetic clocks show statistically significant associations and all four clocks show a consistent direction of effect. **Analyses were restricted to participants with plausible total energy intake based on Willett cutoffs: 500–3,500 kcal/day for women and 800–4,200 kcal/day for men.**

Additional file 1: Table 8a: Association between specific food groups and epigenetic age acceleration (Full adjusted, adjusted for estimated blood cell proportions, **using willet cut offs**)

Specific food groups	Horvath EAA<br>β (95%CI)	Hannum EAA<br>β (95%CI)	GrimAge EAA<br>β (95%CI)	PhenoAge EAA<br>β (95%CI)
**Rural Ghana (n=227)**				
Alcoholic_beverages	**-0.015 (-0.029–-0.001) ***	-0.003 (-0.016–0.009)	-0.001 (-0.009–0.006)	-0.012 (-0.029–0.004)
Cakes_sweets	-0.022 (-0.059–0.015)	-0.034 (-0.069–0.002)	-0.011 (-0.033–0.01)	**-0.054 (-0.098–-0.01) ***
Coffee_tea	0.001 (-0.001–0.003)	0 (-0.002–0.002)	0 (-0.001–0.001)	0.001 (-0.002–0.003)
Condiments	-0.008 (-0.031–0.016)	-0.018 (-0.039–0.003)	**-0.014 (-0.027–-0.001) ***	-0.025 (-0.052–0.002)
Cooking_fats	1.216 (-8.965–11.397)	-3.447 (-12.902–6.008)	-0.167 (-5.946–5.612)	-5.979 (-18.046–6.088)
Dairy_products	-0.01 (-0.03–0.01)	-0.012 (-0.031–0.007)	0.002 (-0.009–0.014)	-0.006 (-0.03–0.018)
Egg	0.022 (-0.048–0.092)	0.02 (-0.045–0.086)	0.016 (-0.024–0.056)	0.055 (-0.029–0.138)
Fermente_maize_products	0 (-0.005–0.005)	0.003 (-0.002–0.008)	0.001 (-0.002–0.004)	0.001 (-0.005–0.007)
Fish	**-0.022 (-0.054–-0.01)***	**-0.013** (-0.043–0.017)	**-0.006** (-0.024–0.012)	**-0.027 (-0.065–-0.01)***
Fruit	0 (-0.004–0.005)	0.001 (-0.003–0.006)	0 (-0.002–0.003)	-0.002 (-0.007–0.004)
Legumes	-0.009 (-0.025–0.008)	0.007 (-0.008–0.023)	0.004 (-0.005–0.014)	0.001 (-0.018–0.021)
Margarine	-0.16 (-0.439–0.119)	-0.119 (-0.379–0.141)	-0.107 (-0.265–0.051)	-0.048 (-0.381–0.285)
Meaty_mixed_dishes	-0.001 (-0.017–0.015)	0.002 (-0.013–0.017)	-0.005 (-0.014–0.004)	0.001 (-0.017–0.02)
Nuts_seeds	0.034 (-0.049–0.118)	-0.016 (-0.093–0.062)	-0.026 (-0.073–0.022)	-0.015 (-0.114–0.085)

Specific food groups	Horvath EAA<br>β (95%CI)	Hannum EAA<br>β (95%CI)	GrimAge EAA<br>β (95%CI)	PhenoAge EAA<br>β (95%CI)
Olive_oil	-0.955 (-5.706–3.797)	-2.408 (-6.815–1.999)	0.619 (-2.077–3.316)	1.192 (-4.452–6.836)
Other_oils	-0.029 (-0.117–0.059)	-0.025 (-0.106–0.057)	-0.022 (-0.072–0.028)	-0.037 (-0.141–0.067)
Palm_oil	0.148 (-0.242–0.538)	0.084 (-0.279–0.446)	-0.103 (-0.324–0.118)	0.082 (-0.382–0.545)
Potatoes	0.022 (-0.017–0.061)	0.005 (-0.031–0.042)	-0.006 (-0.028–0.017)	0.013 (-0.033–0.06)
Poultry	0.023 (-0.022–0.068)	-0.009 (-0.051–0.033)	0.002 (-0.024–0.027)	0.063 (0.01–0.116) *
Processed_meat	0.015 (-0.078–0.107)	-0.036 (-0.122–0.05)	-0.048 (-0.1–0.004)	-0.007 (-0.117–0.102)
Red_meat	-0.002 (-0.027–0.024)	-0.009 (-0.032–0.015)	-0.001 (-0.015–0.013)	0.002 (-0.028–0.032)
Rice_pasta	-0.002 (-0.011–0.007)	0.001 (-0.008–0.009)	-0.001 (-0.007–0.004)	-0.002 (-0.013–0.009)
Roots_tubers_plantain	0.001 (-0.002–0.004)	0.001 (-0.002–0.004)	-0.001 (-0.002–0.001)	0 (-0.004–0.004)
Sodas_juices	-0.002 (-0.006–0.002)	-0.003 (-0.007–0.001)	0 (-0.002–0.003)	-0.002 (-0.007–0.003)
Sweet_spreads	-1.114 (-3.483–1.254)	-0.659 (-2.864–1.546)	-0.017 (-1.364–1.33)	0.69 (-2.128–3.508)
Vegetable_soups_stews_sauces	0 (-0.003–0.002)	0 (-0.003–0.002)	0.001 (-0.001–0.002)	0 (-0.003–0.003)
Vegetables	0 (-0.007–0.008)	0 (-0.007–0.007)	0.002 (-0.003–0.006)	-0.001 (-0.01–0.008)
Vegetarian_mixed_dishes	-0.021 (-0.065–0.022)	-0.025 (-0.065–0.016)	-0.012 (-0.036–0.013)	-0.049 (-0.1–0.003)
White_bread_cereals	-0.002 (-0.013–0.008)	0.001 (-0.009–0.01)	-0.001 (-0.007–0.005)	-0.008 (-0.02–0.004)
Whole_grain_bread_cereals	0.001 (-0.023–0.025)	0 (-0.022–0.022)	-0.001 (-0.014–0.012)	0.005 (-0.023–0.033)
**Urban Ghana (n=202)**				
Alcoholic_beverages	0.004 (-0.044–0.052)	-0.007 (-0.048–0.033)	0.009 (-0.021–0.04)	0.056 (-0.001–0.112)
Cakes_sweets	-0.023 (-0.07–0.024)	-0.018 (-0.057–0.022)	0.014 (-0.016–0.043)	**-0.065 (-0.119–-0.01) ***

Specific food groups	Horvath EAA<br>β (95%CI)	Hannum EAA<br>β (95%CI)	GrimAge EAA<br>β (95%CI)	PhenoAge EAA<br>β (95%CI)
Coffee_tea	0 (-0.003–0.002)	0 (-0.001–0.002)	0 (-0.002–0.001)	-0.002 (-0.005–0)
Condiments	0.001 (-0.019–0.02)	0.002 (-0.014–0.019)	0.006 (-0.007–0.018)	-0.007 (-0.03–0.017)
Cooking_fats	-13.656 (-32.311–4.999)	-15.086 (-30.718–0.545)	-5.342 (-17.05–6.37)	-23.033 (-44.883–1.183)
Dairy_products	0.001 (-0.009–0.012)	-0.001 (-0.01–0.008)	0.006 (-0.001–0.013)	-0.012 (-0.024–0.001)
Egg	-0.04 (-0.095–0.015)	-0.039 (-0.085–0.008)	-0.007 (-0.041–0.028)	-0.061 (-0.126–0.004) *
Fermente_maize_products	**-0.001** (-0.005–0.003)	**-0.001 (-0.005–-0.003)***	**-0.003 (-0.005–-0.003) ***	**-0.003 (-0.009–-0.002)***
Fish	0.011 (-0.008–0.03)	0.008 (-0.008–0.024)	0 (-0.012–0.012)	-0.018 (-0.04–0.005)
Fruit	0.002 (-0.003–0.007)	0.002 (-0.003–0.006)	-0.002 (-0.005–0.002)	0 (-0.007–0.006)
Legumes	-0.002 (-0.02–0.017)	-0.006 (-0.022–0.009)	0 (-0.012–0.011)	0.01 (-0.012–0.031)
Margarine	-0.004 (-0.22–0.211)	-0.064 (-0.245–0.117)	0.114 (-0.019–0.248)	-0.187 (-0.44–0.065)
Meaty_mixed_dishes	0.007 (-0.009–0.023)	0.002 (-0.011–0.015)	0.008 (-0.002–0.018)	0.008 (-0.011–0.026)
Nuts_seeds	0.043 (-0.021–0.107)	0.067 (0.014–0.12)	0.039 (-0.001–0.079)	0.063 (-0.012–0.138)
Olive_oil	0.232 (-0.685–1.149)	-0.14 (-0.912–0.632)	-0.265 (-0.838–0.308)	-0.521 (-1.6–0.558)
Other_oils	-0.063 (-0.123–-0.002) *	-0.038 (-0.09–0.013)	-0.007 (-0.046–0.031)	-0.007 (-0.079–0.066)
Palm_oil	-0.12 (-0.446–0.207)	-0.08 (-0.355–0.195)	0.014 (-0.19–0.219)	-0.203 (-0.587–0.181)
Potatoes	-0.019 (-0.047–0.009)	0.002 (-0.021–0.026)	0.01 (-0.008–0.028)	-0.001 (-0.034–0.033)
Poultry	-0.001 (-0.041–0.038)	-0.002 (-0.035–0.032)	0.009 (-0.016–0.033)	-0.01 (-0.056–0.037)
Processed_meat	-0.016 (-0.061–0.03)	0.009 (-0.029–0.048)	**0.03 (0.001–0.058) ***	0.013 (-0.041–0.067)
Red_meat	-0.006 (-0.027–0.016)	**-0.019 (-0.037–-0.001) ***	0.011 (-0.002–0.025)	-0.011 (-0.037–0.014)

Specific food groups	Horvath EAA<br>β (95%CI)	Hannum EAA<br>β (95%CI)	GrimAge EAA<br>β (95%CI)	PhenoAge EAA<br>β (95%CI)
Rice_pasta	0.002 (-0.007–0.01)	0.001 (-0.006–0.008)	0.004 (-0.001–0.009)	0.002 (-0.008–0.012)
Roots_tubers_plantain	0 (-0.006–0.006)	0.003 (-0.002–0.009)	0 (-0.004–0.004)	-0.002 (-0.009–0.006)
Sodas_juices	0.006 (0.001–0.01) *	0 (-0.003–0.004)	-0.001 (-0.004–0.002)	0 (-0.005–0.005)
Sweet_spreads	0.215 (-0.187–0.616)	**0.379 (0.045–0.713) ***	-0.168 (-0.418–0.083)	0.111 (-0.363–0.585)
Vegetable_soups_stews_sauces	-0.001 (-0.002–0.001)	0 (-0.002–0.001)	-0.001 (-0.002–0)	0.001 (-0.001–0.003)
Vegetables	0.001 (-0.006–0.008)	0.002 (-0.004–0.008)	0 (-0.004–0.004)	-0.002 (-0.01–0.006)
Vegetarian_mixed_dishes	0.02 (-0.022–0.061)	0.001 (-0.034–0.036)	0.012 (-0.014–0.038)	0.022 (-0.027–0.071)
White_bread_cereals	0.003 (-0.006–0.011)	0.004 (-0.003–0.011)	0 (-0.006–0.005)	0.003 (-0.007–0.013)
Whole_grain_bread_cereals	0.002 (-0.01–0.014)	-0.001 (-0.011–0.01)	0.008 (0–0.015) *	0 (-0.014–0.014)
**Amsterdam (Ghanaians, n=201)**				
Alcoholic_beverages	-0.002 (-0.006–0.001)	0 (-0.002–0.003)	0.001 (-0.001–0.002)	0 (-0.004–0.004)
Cakes_sweets	**-0.061 (-0.109–-0.013) ***	-0.015 (-0.051–0.022)	0.007 (-0.017–0.03)	-0.042 (-0.09–0.005)
Coffee_tea	0 (-0.001–0.001)	0 (-0.001–0.001)	0 (0–0.001)	0 (-0.001–0.001)
Condiments	-0.001 (-0.016–0.014)	0.008 (-0.003–0.019)	-0.004 (-0.012–0.003)	0.011 (-0.003–0.026)
Cooking_fats	-1.376 (-4.636–1.884)	-1.113 (-3.545–1.319)	**-2.634 (-4.184–-1.084) ***	-2.084 (-5.295–1.128)
Dairy_products	-0.004 (-0.013–0.005)	-0.002 (-0.008–0.005)	0.001 (-0.003–0.006)	0.002 (-0.007–0.011)
Egg	-0.067 (-0.131– 0.004)	-0.035 (-0.082–0.013)	-0.012 (-0.043–0.02)	**-0.064 (-0.126–-0.001) ***
Fermente_maize_products	-0.003 (-0.014–0.009)	0.003 (-0.006–0.012)	0 (-0.006–0.006)	-0.002 (-0.013–0.01)
Fish	0.011 (-0.012–0.035)	0.005 (-0.013–0.023)	0.002 (-0.01–0.013)	0.012 (-0.012–0.035)

Specific food groups	Horvath EAA<br>β (95%CI)	Hannum EAA<br>β (95%CI)	GrimAge EAA<br>β (95%CI)	PhenoAge EAA<br>β (95%CI)
Fruit	-0.001 (-0.003–0.002)	-0.001 (-0.003–0.001)	-0.001 (-0.002–0)	0.001 (-0.002–0.004)
Legumes	-0.001 (-0.027–0.024)	**-0.022 (-0.041–-0.002) ***	-0.001 (-0.014–0.012)	-0.005 (-0.031–0.02)
Margarine	-0.002 (-0.177–0.173)	-0.002 (-0.132–0.129)	0.025 (-0.061–0.11)	-0.063 (-0.235–0.11)
Meaty_mixed_dishes	0.006 (-0.015–0.027)	0.009 (-0.007–0.024)	-0.006 (-0.016–0.004)	0.008 (-0.013–0.028)
Nuts_seeds	-0.007 (-0.054–0.04)	0.001 (-0.034–0.037)	0.002 (-0.021–0.025)	0 (-0.046–0.047)
Olive_oil	-0.035 (-0.444–0.375)	-0.036 (-0.342–0.27)	-0.089 (-0.289–0.111)	-0.202 (-0.605–0.201)
Other_oils	-0.025 (-0.075–0.025)	0 (-0.038–0.037)	-0.005 (-0.03–0.02)	0.01 (-0.04–0.059)
Palm_oil	-0.891 (-2.043–0.262)	-0.352 (-1.216–0.513)	0.466 (-0.098–1.029)	-1.026 (-2.161–0.11)
Potatoes	-0.004 (-0.023–0.015)	-0.007 (-0.021–0.007)	0.002 (-0.007–0.011)	0.001 (-0.017–0.02)
Poultry	-0.007 (-0.039–0.025)	0.007 (-0.017–0.031)	-0.015 (-0.03–0.001)	0.01 (-0.021–0.042)
Processed_meat	-0.034 (-0.079–0.011)	-0.009 (-0.043–0.025)	0.002 (-0.02–0.025)	**-0.052 (-0.096–-0.007) ***
Red_meat	0 (-0.027–0.027)	0.007 (-0.013–0.027)	-0.009 (-0.022–0.004)	-0.017 (-0.043–0.01)
Rice_pasta	-0.004 (-0.013–0.006)	0.003 (-0.004–0.01)	-0.001 (-0.006–0.003)	0.002 (-0.008–0.011)
Roots_tubers_plantain	-0.002 (-0.009–0.004)	-0.001 (-0.006–0.004)	0 (-0.003–0.003)	-0.003 (-0.01–0.004)
Sodas_juices	-0.001 (-0.004–0.002)	0 (-0.003–0.002)	0 (-0.001–0.002)	-0.002 (-0.005–0.002)
Sweet_spreads	-0.032 (-0.21–0.147)	-0.035 (-0.168–0.098)	0.058 (-0.029–0.144)	0.032 (-0.144–0.208)
Vegetable_soups_stews_sauces	0 (-0.002–0.003)	0 (-0.002–0.001)	0.001 (0–0.002)	0.001 (-0.001–0.003)
Vegetables	-0.001 (-0.006–0.004)	-0.001 (-0.005–0.003)	0 (-0.003–0.002)	-0.001 (-0.006–0.005)
Vegetarian_mixed_dishes	0.03 (-0.014–0.074)	0.023 (-0.01–0.056)	-0.002 (-0.024–0.019)	0.036 (-0.008–0.079)

Specific food groups	Horvath EAA<br>β (95%CI)	Hannum EAA<br>β (95%CI)	GrimAge EAA<br>β (95%CI)	PhenoAge EAA<br>β (95%CI)
White_bread_cereals	-0.004 (-0.015–0.006)	-0.007 (-0.014–0.001)	0 (-0.005–0.005)	0.003 (-0.008–0.013)
Whole_grain_bread_cereals	-0.008 (-0.024–0.008)	0.001 (-0.011–0.013)	0 (-0.008–0.008)	0 (-0.016–0.017)

This table presents the associations between intake of specific food groups (grams/day) and four measures of epigenetic age acceleration (Horvath EAA, Hannum EAA, GrimAge EAA, and PhenoAge EAA), stratified by study site (rural Ghana, urban Ghana, and Amsterdam). **Beta coefficients (β) and 95% confidence intervals (CI)** are derived from linear regression models fully **adjusted** for age, sex, education level, smoking status, physical activity, BMI, alcohol intake, diabetes status, and hypertension status. Positive β values indicate greater epigenetic age acceleration, while negative values suggest slower biological aging relative to chronological age. Asterisks (*) denote statistically significant associations based on the 95% confidence interval. Bolded estimates indicate a consistent direction of association across all three study sites. Overall significance is determined using the "2+ rule": an association is considered robust if at least two clocks show statistically significant associations and all four clocks show a consistent direction of effect. Analyses were restricted to participants with plausible dietary intake based on Willett cut-offs (500–3500 kcal/day for women and 800–4200 kcal/day for men).

Additional file 1: Table 8b: Fully Adjusted Associations Between Dietary Patterns, Plant-Based and Animal-Based Food Proportions, and Epigenetic Age Acceleration Among Ghanaian Adults, Stratified by Study Site, **adjusted for estimated blood cell proportions** (**used willet cut-offs**).

Specific food groups	Horvath EAA<br>Adjusted β (95%CI)	Hannum EAA<br>Adjusted β (95%CI)	GrimAge EAA<br>Adjusted β (95%CI)	PhenoAge EAA<br>Adjusted β (95%CI)
**Data driven dietary patterns**				
**Rural Ghana (n=227)**				
DP1 (Plant-based & mixed traditional foods)	0.122 (-1.368–1.611)	0.237 (-1.147–1.622)	0.102 (-0.743–0.947)	-0.366 (-2.135–1.402)
DP2 (Animal-based and fried/staple-rich foods)	-0.464 (-1.537–0.609)	-0.86 (-1.852–0.132)	-0.405 (-1.013–0.202)	-0.408 (-1.683–0.867)
DP3 (Modern/processed foods)	-0.195 (-1.128–0.737)	0.458 (-0.407–1.324)	-0.085 (-0.615–0.444)	-0.422 (-1.529–0.685)
**Urban Ghana (n=202)**				
DP1 (Plant-based & mixed traditional foods)	0.506 (-0.752–1.763)	0.962 (-0.089–2.012)	0.058 (-0.73–0.846)	-0.068 (-1.552–1.417)
DP2 (Animal-based and fried/staple-rich foods)	-0.145 (-1.015–0.725)	-0.477 (-1.206–0.252)	**0.641 (0.105–1.178) ***	-0.682 (-1.702–0.339)
DP3 (Modern/processed foods)	0.296 (-0.754–1.347)	0.178 (-0.706–1.063)	0.028 (-0.63–0.686)	0.027 (-1.212–1.267)
**Amsterdam (Ghanaians, n=201)**				
DP1 (Plant-based & mixed traditional foods)	-0.297 (-1.121–0.528)	-0.209 (-0.825–0.406)	0.011 (-0.393–0.415)	0.385 (-0.428–1.198)
DP2 (Animal-based and fried/staple-rich foods)	-0.343 (-1.197–0.511)	0.219 (-0.419–0.857)	-0.209 (-0.626–0.209)	-0.495 (-1.338–0.347)
DP3 (Modern/processed foods)	-0.019 (-1.218–1.179)	-0.141 (-1.035–0.753)	0.146 (-0.44–0.732)	0.563 (-0.618–1.744)
**Proportion of plant/animal-based food intake**				
**Rural Ghana (n=227)**				

Specific food groups	Horvath EAA<br>Adjusted β (95%CI)	Hannum EAA<br>Adjusted β (95%CI)	GrimAge EAA<br>Adjusted β (95%CI)	PhenoAge EAA<br>Adjusted β (95%CI)
Proportion healthful plant-based	0.026 (-0.044–0.096)	0.019 (-0.047–0.084)	-0.005 (-0.045–0.034)	-0.003 (-0.086–0.081)
Proportion unhealthful plant-based	-0.012 (-0.09–0.065)	0.015 (-0.057–0.087)	-0.005 (-0.049–0.039)	-0.011 (-0.103–0.081)
Proportion animal-based	-0.008 (-0.072–0.056)	-0.014 (-0.074–0.046)	0.016 (-0.021–0.052)	0.022 (-0.055–0.098)
**Urban Ghana (n=202)**				
Proportion healthful plant-based	0.021 (-0.057–0.099)	0.044 (-0.021–0.109)	0.031 (-0.017–0.08)	0.004 (-0.088–0.095)
Proportion unhealthful plant-based	0.022 (-0.061–0.105)	-0.011 (-0.081–0.059)	-0.025 (-0.077–0.027)	-0.051 (-0.149–0.046)
Proportion animal-based	-0.024 (-0.084–0.037)	-0.021 (-0.072–0.03)	-0.01 (-0.048–0.028)	0.032 (-0.039–0.104)
**Amsterdam (Ghanaians, n=201)**				
Proportion healthful plant-based	-0.001 (-0.066–0.064)	-0.005 (-0.054–0.043)	-0.027 (-0.059–0.005)	-0.012 (-0.076–0.053)
Proportion unhealthful plant-based	-0.028 (-0.116–0.059)	0.006 (-0.059–0.072)	0.006 (-0.037–0.048)	-0.021 (-0.107–0.066)
Proportion animal-based	0.019 (-0.043–0.081)	-0.007 (-0.053–0.039)	0.026 (-0.004–0.056)	0.018 (-0.043–0.079)

This table presents the associations between data-driven dietary patterns, proportions of plant-based and animal-based food intake, and four measures of epigenetic age acceleration (Horvath EAA, Hannum EAA, GrimAge EAA, and PhenoAge EAA), stratified by study site (rural Ghana, urban Ghana, and Amsterdam). The values shown are beta coefficients (β) and 95% confidence intervals (CI), derived from linear regression models. Associations are shown after full adjustment (in brackets) for age, sex, education level, smoking status, physical activity, BMI, alcohol intake, diabetes status, and hypertension status. Positive β values indicate greater epigenetic age acceleration, while negative values reflect slower biological aging relative to chronological age. Asterisks (*) indicate statistically significant associations based on the 95% confidence interval. Bolded estimates indicate a consistent direction of association across all three study sites. Overall significance is determined using the "2+ rule," whereby an association is considered robust if at least two epigenetic clocks show statistically significant associations and all four clocks show a consistent direction of effect. **Analyses were restricted to participants with plausible total energy intake based on Willett cutoffs: 500–3,500 kcal/day for women and 800–4,200 kcal/day for men.**

Additional file 1: Table 9. Mediation of the Association Between Diet and Epigenetic Age Acceleration by one carbon metabolism and Inflammation proxies, adjusted for cell-type

Dietary Factor	Analysis Type	Mediator	ACME (Indirect)	ADE (Direct)	Total Effect	Proportion Mediated (%)
**Rural Ghana**						
Fish Intake	PhenoAge EAA	Vitamin B9	0.0027 (-0.0034– 0.0103)	-0.0336 (-0.0696– 0.0057)	-0.0309 (-0.067– 0.0089)	8.63%
		Vitamin B12	0.0036 (-0.0069– 0.0143)	-0.0346 (-0.075– 0.0089)	-0.0309 (-0.067– 0.0089)	11.77%
		Vitamin D	-0.0143 (-0.0439– 0.0229)	-0.0166 (-0.0701– 0.0357)	-0.0309 (-0.067– 0.0089)	**46.27%**
		CRP	-0.0006 (-0.0032– 0.002)	-0.0304 (-0.0671– 0.0092)	-0.0309 (-0.067– 0.0089)	1.85%
Fish intake	Horvath EAA	Vitamin B9	0.0003 (-0.0053– 0.0063)	-0.0291 (-0.0575– 6)	-0.0288 (-0.0592– 0.0019)	1.18%
		Vitamin B12	-0.0048 (-0.0146– 0.0032)	-0.024 (-0.0539– 0.0081)	-0.0288 (-0.0592– 0.0019)	**16.58%**
		Vitamin D	0.0056 (-0.0243– 0.0385)	-0.0344 (-0.0781– 0.0065)	-0.0288 (-0.0592– 0.0019)	**19.45%**
		CRP	0.0003 (-0.0053– 0.0063)	-0.0291 (-0.0575– 0.0006)	-0.0288 (-0.0592– 0.0019)	1.18%
**Urban Ghana**						
Fermented Products	PhenoAge EAA	Vitamin B9	-0.0005 (-0.0018– 0.0005)	-0.005 (-0.0097– 0.0001)	-0.0055 (-0.0102– -0.0005)	9.13%
		Vitamin B12	0 (-0.0005– 0.0003)	-0.0055 (-0.0102– -0.0004)	-0.0055 (-0.0102– -0.0005)	0.83%
		Vitamin D	-0.0002 (-0.0014– 0.0006)	-0.0053 (-0.0104– 0.0001)	-0.0055 (-0.0102– -0.0005)	3.75%
		CRP	-0.0001 (-0.001– 0.0001)	-0.0051 (-0.0097– 0.0006)	-0.0053 (-0.01– 0.0002)	2.63%
Fermented Products	Hannum EAA	Vitamin B9	0.0001 (-0.0005– 0.0012)	-0.002 (-0.0051– 0.0027)	-0.0018 (-0.0048– 0.0029)	7.56%
		Vitamin B12	0 (-0.0003– 2)	-0.0018 (-0.0048– 0.0029)	-0.0018 (-0.0048– 0.0029)	0.6%
		Vitamin D	0.0001 (-0.0003– 0.0006)	-0.0019 (-0.0048– 0.0027)	-0.0018 (-0.0048– 0.0029)	4.06%
		CRP	0.9546 (-3.3446– 3.1369)	-24.8325 (-40.1663– 3.7054)	-23.8779 (-37.9729– 2.5773)	4%
**Amsterdam (Ghanaians)**						
Condiments	Hannum EAA	Vitamin B9	0.001 (-0.0016– 0.0039)	0.0085 (-0.0024– 0.0207)	0.0096 (-0.0008– 0.0204)	**10.85%**
		Vitamin B12	-0.0004 (-0.007– 0.006)	0.0099 (-0.0017– 0.0216)	0.0096 (-0.0008– 0.0204)	3.68%
		Vitamin D	0.0003 (-0.0009– 0.0019)	0.0093 (-0.0012– 0.0206)	0.0096 (-0.0008– 0.0204)	3.13%
		CRP	0.0001 (-0.0011– 0.0013)	0.0093 (-0.0011– 0.0213)	0.0094 (-0.0013– 0.0216)	1.07%
Condiments	PhenoAge EAA	Vitamin B9	0.0022 (-0.0008– 0.006)	0.0101 (-0.0032– 0.0231)	0.0123 (-0.0005– 0.0246)	**17.87%**
		Vitamin B12	-0.0006 (-0.0095– 0.0089)	0.0129 (-0.0028– 0.028)	0.0123 (-0.0005– 0.0246)	4.65%
		Vitamin D	0.0007 (-0.0007– 0.003)	0.0117 (-0.0011– 0.024)	0.0123 (-0.0005– 0.0246)	5.44%
		CRP	-0.0003 (-0.0023– 0.0013)	0.0135 (0.0003– 0.0268)	0.0132 (0.0001– 0.0267)	2.46%

Mediation analysis results showing the indirect effects (ACME), direct effects (ADE), total effects, and proportion mediated (%) of selected micronutrients and CRP in the association between dietary factors and epigenetic age acceleration (EAA), based on two clocks: Hannum and PhenoAge. Analyses were conducted among Ghanaians living in rural Ghana, urban Ghana, and Amsterdam.
Mediators were selected based on prior biological relevance to one-carbon metabolism (e.g., Vitamin B9, B12), inflammation (CRP), and antioxidant pathways (e.g., Vitamin D). Mediation was evaluated only where statistically significant associations between diet and EAA were observed.
A higher proportion mediated (%) indicates a greater contribution of the mediator to the total effect. **Bolded rows** indicate mediation pathways where ≥10% of the total effect was mediated.
All mediation analyses were performed using the mediation package in R, based on a counterfactual framework for causal mediation analysis.
